# Concentration and chemical form of dietary zinc shape the porcine colon microbiome, its functional capacity and antibiotic resistance gene repertoire

**DOI:** 10.1038/s41396-020-0730-3

**Published:** 2020-08-03

**Authors:** Robert Pieper, Temesgen H. Dadi, Laura Pieper, Wilfried Vahjen, André Franke, Knut Reinert, Jürgen Zentek

**Affiliations:** 1 Institute of Animal Nutrition, Department of Veterinary Medicine, Freie Universität Berlin, Berlin, Germany; 2 Department of Mathematics and Computer Science, Institute of Computer Science, Freie Universität Berlin, Berlin, Germany; 3 Max Planck Institute for Molecular Genetics, Berlin, Germany; 4 Institute for Veterinary Epidemiology and Biostatistics, Department of Veterinary Medicine, Freie Universität Berlin, Berlin, Germany; 5 Institute of Clinical Molecular Biology, Christian-Albrechts-University of Kiel, University Hospital Schleswig Holstein, Kiel, Germany

**Keywords:** Microbiome, Applied microbiology

## Abstract

Despite a well-documented effect of high dietary zinc oxide on the pig intestinal microbiota composition less is it yet known about changes in microbial functional properties or the effect of organic zinc sources. Forty weaning piglets in four groups were fed diets supplemented with 40 or 110 ppm zinc as zinc oxide, 110 ppm as Zn-Lysinate, or 2500 ppm as zinc oxide. Host zinc homeostasis, intestinal zinc fractions, and ileal nutrient digestibility were determined as main nutritional and physiological factors putatively driving colon microbial ecology. Metagenomic sequencing of colon microbiota revealed only clear differences at genus level for the group receiving 2500 ppm zinc oxide. However, a clear group differentiation according to dietary zinc concentration and source was observed at species level. Functional analysis revealed significant differences in genes related to stress response, mineral, and carbohydrate metabolism. Taxonomic and functional gene differences were accompanied with clear effects in microbial metabolite concentration. Finally, a selection of certain antibiotic resistance genes by dietary zinc was observed. This study sheds further light onto the consequences of concentration and chemical form of dietary zinc on microbial ecology measures and the resistome in the porcine colon.

## Introduction

Zinc is an essential trace element, involved in manifold metabolic processes in the body [1]. In practical diets for pigs, zinc must be supplemented in small amounts, (50–80 ppm), in order to meet the animals requirements and prevent zinc deficiency [2, 3]. Despite the need to supplement zinc to meet the zinc requirements within the legal boundaries (e.g. in the EU, the maximum permitted level for pigs is currently 150 mg zinc/kg diet), very high dietary zinc oxide (ZnO) levels (i.e., 2000–3000 mg zinc/kg) have been shown to reduce diarrhea and improve growth performance in weaning pigs and are thus often used as effective alternative to antimicrobial growth promoters [4, 5]. Proposed mechanisms include alteration of the intestinal microbiota composition and activity, metabolic reactions in the liver, pancreas, and small intestinal tissue such as barrier function and secretory reactions [6–9]. On the other hand, the use of such high amounts of dietary zinc is regarded more and more critically due to high zinc concentrations in manure that may increase soil zinc concentrations and because of possible co-selection for antibiotic resistance among bacteria in the pig gut [10, 11]. In the past years, organic zinc sources including certain chelates of amino acids such as glycine, lysine, or methionine have been authorized in the EU as feed additives. Although it has been often postulated that organic zinc source may have a higher bioavailability as compared with the inorganic sources such as ZnO or ZnSO_4_, results in the literature are sometimes not as clear [12]. Furthermore, and in contrast to studies with very high dietary ZnO, little is yet known about the influence of inorganic and organic zinc sources at levels meeting animal requirements, on the intestinal microbiota composition and function as well as putative effects on antibiotic resistance genes in pigs.

The current study was thus conducted to compare the effect of low, normal, or very high dietary levels of dietary ZnO with a diet containing a normal level of an organic zinc source on zootechnical traits, body zinc status as indicator of bioavailability, and the composition and function of the intestinal microbiome in weaned piglets. It was hypothesized that the chemical form of the zinc source or low concentration of dietary ZnO would differentially affect the intestinal microbiome as compared with very high dietary ZnO due to effects on host zinc homeostasis as well as direct Zn-dependent effects on the intestinal microbiota.

## Material and methods

### Experimental procedures

#### Animals and housing

A total of 40 (m/f) Landrace piglets, weaned at the age of 25 ± 1 days with a mean body weight (BW) of 6.0 ± 0.3 kg, were placed in commercial flat deck pens (*n* = 2 piglets/pen) balancing for gender and BW. Pens were randomly assigned to one of four groups. Water and feed were provided ad libitum. The room temperature was maintained at 26 °C on the day of weaning and reduced at regular intervals to achieve 22 °C during the first week. Feed intake and BW of pigs were recorded weekly. Fecal consistency was scored daily based on a scale between 1 = entirely liquid via 3 = normal, soft and formed feces to 5 = dry, hard pellets.

#### Diets and diet analyses

Four diets (Supplementary Table S1) were formulated to provide different dietary concentrations of zinc from either ZnO (Sigma, Taufkirchen, Germany) or zinc lysinate chelate (i.e. zinc chelate of bislysinate HCl, CCDC deposition #1048236, registration #3b613 according to Reg (EG) 1831/2003). Diets were formulated to meet or exceed nutrient requirements of weaning piglets [2]. Basal dietary zinc level was supplemented with either 40 or 110 ppm ZnO (40ZnO and 110ZnO) or 110 ppm Zn-Lysinate (adjusted for total Lysine; 110Zn-Lys), or with 2500 ppm with ZnO (2500ZnO). Titanium dioxide was added to the complete diets to allow determination of nutrient digestibility. Piglets were fed the respective diets for 3 weeks before euthanasia and subsequent sampling of gut contents. Proximate nutrients (ash, crude fiber, crude protein, ether extract), starch, and (trace) elements in the diets and digesta were determined using standard procedures. Titanium content in the diets and the digesta was measured as described [13].

#### Sampling

Piglets were euthanized on experimental day 21 ± 1 by intracardial injection of T61® (Intervet, Unterschleißheim, Germany) after sedation with ketamine hydrochloride (Ursotamin®, Serumwerk Bernburg AG, Germany) and azaperone (Stresnil®, Jansen-Cilag, Neuss, Germany). Pigs were euthanized 4 h (±10 min) after the morning meal to minimize bias due to different gut filling and supply of substrate for microbial fermentation. Following euthanasia, ileum and colon contents as well as tissue samples from jejunum, liver, spleen, kidney, bone (metacarpal IV), and pancreas were taken and immediately stored at −80 °C until further analysis.

### Nutrient digestibility

Apparent ileal digestibility of dietary matter, crude protein, ether extract, starch, and zinc were calculated for all piglets (*n* = 10/group) as follows:

$${\mathrm{Apparent}}\;{\mathrm{ileal}}\;{\mathrm{digestibility}}\left( \% \right) = 1 - \left[ {\left( {{\mathrm{Ti}}_F \times {\mathrm{N}}_D} \right)/\left( {{\mathrm{Ti}}_D \times {\mathrm{N}}_F} \right)} \right] \times \;100,$$

where N_*F*_ is the concentration of nutrients in the diet, N_*D*_ is the concentration of nutrients in ileal digesta, Ti_*F*_ is the concentration of titanium in the diet, and Ti_*D*_ the concentration of titanium in ileal digesta.

### Measures of host zinc homeostasis

#### Colon luminal zinc fractions

Intestinal zinc fractions (i.e., defined here as total zinc, free zinc ions, protein-bound zinc) were determined for all piglets as described [14]. Briefly, samples were diluted (1:2 vol:vol) in water, homogenized for 1 h at the room temperature and centrifuged at 14,200 × *g* for 10 min. Supernatants were withdrawn quantitatively and applied on polymeric reversed-phase sorbent columns (8B-S100-FBJ, Phenomenex, Aschaffenburg, Germany). The resulting eluents contained the total free inorganic zinc of the samples. Protein-associated zinc was determined from eluents after elution with acetonitrile/water (4:6 vol:vol) and acetonitrile/formic acid (7:3 vol:vol) and evaporation of the organic phase by vacuum centrifugation. Total zinc in untreated digesta and the sample fractions was determined in an atomic absorption spectrometer (AAS vario 6, Analytik Jena, Germany) after total hydrolysis of sample fractions in hydrochloric acid (37%) for 90 min at 250 °C.

#### Organ zinc concentration

Zinc concentration in tissue samples from jejunum, liver, kidney, pancreas, bone (metacarpal IV), and spleen of all animals was determined after dry ashing at 600 °C for 8 h [7]. Zinc concentration was determined by atomic absorption spectrometry in an AAS vario 6 spectrometer (Analytik Jena, Jena, Germany).

#### Expression of host zinc transporters and metallothionein

Expression of genes related to zinc homeostasis was performed for *n* = 6 piglets/group as described previously [15]. Briefly, jejunal tissue was extracted using the Nucleo Spin® kit (Macherey Nagel, Düren, Germany) and equimolar mRNA transcribed into complementary DNA. Subsequently, real-time qPCR was performed using primers targeting zinc transporter (*ZnT1, ZIP4*), and two metallothionein isoforms (*MT-1A, MT-2B*). Relative gene expression was calculated based on PCR efficiency and Ct values of target genes normalized using three housekeeping genes (i.e., Succinate Dehydrogenase subunit A, β-2-microglobulin and β-actin).

### Microbial ecology measures

#### Bacterial metabolites

Bacterial metabolites were analyzed in colon contents of *n* = 6 piglets/group as described [16]. For determination of d- and l-lactate, samples were treated with 0.5 M CuSO_4_ prior to analysis by high performance liquid chromatography on an Agilent 1100 chromatograph equipped with a Phenomenex C18 (4.0 × 2.0 mm) guard column followed by a Phenomenex Chirex 3126 (D)-penicillamine column (150 × 4.6 mm) and a UV detector at 253 nm. The carrier was CuSO_4_ in a gradient from 0.5 to 2.5 mmol/l with a flow rate of 1.0 ml/min at 35 °C and the injection volume was 20 µl. For NH_4_ analysis, 20 µl of a sample was chlorinated with 100 µl of 0.2% alkaline hypochloride (Sigma Aldrich, Deisenhofen, Germany) to convert NH_3_ to chloramine (NH_2_Cl) following reaction with thymol to N-chlor-2-isopropyl-5-methyl chinon-monoimin and further to indophenol using 100 µl of 5% phenol nitroprusside (Sigma Aldrich, Deisenhofen, Germany). Samples were incubated in microtitration plates for 100 min and extinction was measured at 620 nm in a Tecan Sunrise^TM^ microplate reader (Tecan Austria GmbH, Grödig, Austria). SCFA were determined by gas chromatography on an Agilent 6890 gas chromatography system with flame ionization detector and autosampler (Agilent Technologies, Böblingen, Germany). Digesta samples were acidified with oxalic acid, centrifuged for 3 min at 14,000 *g* followed by addition of the internal standard (caproic acid). Individual SCFA were separated on a polyethylene glycol column (30 m by 530 µm by 1.00 µm; HP-INNOWax). Hydrogen was used as carrier gas and the injection volume was 1 µl. The flow rates of hydrogen and air were 20 and 400 ml/min, respectively. The initial oven temperature was 70 °C, followed by an increase at a rate of 15 °C/min and then a final temperature of 190 °C for 4 min.

#### Metagenomic sequencing and bioinformatics

Total genomic DNA was extracted from colon digesta of *n* = 6 piglets/group using the QIAamp Fast DNA Stool Mini Kit (Qiagen, Hilden, Germany). After DNA quality control using the Genomic DNA Analysis ScreenTape on a 2200 TapeStation Instrument (Agilent Technologies) and concentration measurement using the Qubit dsDNA BR Assay Kit (Thermo Fisher Scientific), shotgun metagenomic libraries were generated using the Nextera DNA Library Preparation Kit (Illumina) according to the manufacturer’s instructions. Libraries were quality-controlled using the D5000 DNA Analysis ScreenTape on a 2200 TapeStation Instrument (Agilent Technologies), and sequenced on an Illumina NextSeq500 with 2 × 150 bp. Finally, sequencing reads were demultiplexed based on the used Nextera indices (dual indexing principle).

#### Bacterial taxonomic analysis

An average of 12,396,718 ± 2,392,293 sequences per sample was obtained ranging from 6,854,414 to 16,794,567. The FLEXBAR tool [17] was used to remove adapter sequences from the metagenomic reads and discard reads with length shorter than 100 nucleotides. We mapped quality checked sequences against reference genomes of 13,193 different bacterial and archaeal species taken from NCBI (ftp://ftp.ncbi.nlm.nih.gov/genomes/genbank/). Further downstream analysis was based on the mapping results produced by yara [18]. The resulting mapping files were further analyzed using the Species-Level Identification of Microorganisms from Metagenomes (SLIMM) tool [19] to produce for taxonomic profiles of each sample. We calculated relative abundances of individual taxa using the SLIMM tool. SLIMM defines relative abundance as the number of reads assigned uniquely to a species divided by the total number of mapped reads then corrected for genome sequence length. A relative abundance cut-off of 0.01% was used to consider a given taxon as present.

#### Functional analysis of metagenomic reads

For functional analysis of metagenomic reads, we used the EBI Metagenomics resource (https://www.ebi.ac.uk/metagenomics/; [20]). Briefly, after quality control of initial reads and separation of rRNA coding sequences, the pipeline predicts protein coding sequences and protein family membership using the InterPro database. InterPro matches are then associated with Gene ontology (GO) terms as a method to identify relevant groups of genes that function together. GOs are summarized under three aspects: molecular function (molecular activities of gene products), cellular component (where gene products are active), and biological process (pathways and larger processes of multiple gene products) and ordered hierarchically, with less specific parent (or GO slim) terms providing an overview of the GO content, and more specific GO child terms. The relative abundances of GO slim and associated GO terms were used for further analysis.

#### Resistome analysis

For resistome analysis, we mapped metagenomic reads against version 1.1.2 of the Comprehensive Antibiotic Resistance Database (CARD; http://arpcard.mcmaster.ca; [21]) using yara read mapper as described above. Then we used the number of reads mapped to each resistance gene per 1000 bp as an indicator for the relative sequence abundance for each gene. Only genes coding for a true resistance were used for further analysis, whereas genes related to multidrug resistance, transcription factors, or regulating factors were deleted from the initial list. Within AR genes coding for β-lactam resistance, ‘*bla*_*CTX-M*_-type’ and ‘*bla*_*TEM*_-like’ genes were summarized as one group, whereas the genes related to vancomycin (glycopeptide) resistance were summarized under the term ‘vanB gene cluster’.

### Statistical analyses

Differential abundance of bacterial genera between treatments was visualized using the hclust package in R. On the species level, we used a partial least square discriminant analysis (PLS-DA) model to determine the bacterial taxa that contributed to discrimination between experimental groups. The model values (Q2(cum) > 0.6 and R2(Y) > 0.9) were used as indicators of model fit. Following, a variable importance in projection (VIP) values were obtained, where VIP is an estimation of the influence of every bacterial species on the discrimination of *y* variables (experimental groups). Larger VIP values indicate a greater influence of a bacterial species, and VIP values of >1 were regarded as significant. The PLS-DA obtaining of VIP scores was performed with SIMCA-P + (Version 12.0; Umetrics, Umea, Sweden). The 60 species with the highest VIP values were then used for heatmap visualization using the hclust package in R. A Venn diagram illustrating the unique and shared bacterial species among experimental groups was drawn using the online tool ‘Venny 2.1’ (http://bioinfogp.cnb.csic.es/tools/venny/).

Means between treatments were analyzed by ANOVA followed Tukey post hoc test in SPSS (IBM, USA). Generally, *P* < 0.05 was considered significant.

## Results

### Animal performance, nutrient digestibility, and intestinal and body zinc status

No clinical signs of disease were observed and diarrhea (i.e., defined as score <2 for 2 consecutive days) was only observed sporadically without any group effects. Feed intake and growth was low during the first experimental week. However, significantly higher feed intake was observed for 2500ZnO and 110ZnLys groups (443 ± 35 and 423 ± 16 g/day, respectively) as compared with 40ZnO and 110ZnO groups (323 ± 18 and 401 ± 20 g/day, respectively) during the second experimental week. Concomitantly, average daily gain was higher in the 2500ZnO and 110ZnLys groups (271 ± 21 and 257 ± 19 g/day, respectively) as compared with 40ZnO and 110ZnO groups (186 ± 29 and 238 ± 17 g/day, respectively) during this period. No significant differences between groups were observed thereafter until the end of the experimental period. Similarly, feed:gain ratio was not different during the entire period (data not shown). Ileal apparent nutrient digestibility (i.e., organic matter, crude protein, ether extract, starch) did not differ between groups (Supplementary Table S2). Similarly, apparent ileal digestibility of zinc did not differ but was numerically lower in the 2500ZnO group. Pigs in the 2500ZnO group had significantly higher levels of total, free, and protein-bound zinc fractions in colon digesta as compared with the other groups (Table 1). No differences were observed between the other treatment groups. Similarly, zinc concentration in jejunal tissue, liver, kidney, and pancreas was higher (*P* < 0.05) in 2500ZnO group as compared with the other groups, whereas no clear differences were observed between those (Supplementary Table 3). With regard to measures of intestinal zinc homeostasis, jejunal expression of the *ZIP4* gene was lower (*P* < 0.05) in 2500ZnO group as compared with the other dietary treatments (Supplementary Table S4). In contrast, highest expression was observed in the 2500ZnO group for *ZnT1, MT1A*, and *MT2B* genes (*P* < 0.05). No other differences were observed between the other groups, except for higher expression of *ZnT1* gene in 110ZnO as compared with 40ZnO group (*P* < 0.05).

**Table 1:** Zinc fractions (total, free, protein-bound) in colon digesta of piglets fed diets with added zinc oxide at 40 ppm (40ZnO), 110 ppm (110ZnO), 2500 ppm (2500ZnO), or 110 ppm Zn-Lysinate (110ZnLys) over a period of 3 weeks.

Item	40ZnO	110ZnO	2500ZnO	110ZnLys	*P* value
ppm dry matter					
Total zinc	506 ± 52^a^	1013 ± 40^a^	11730 ± 862^b^	938 ± 77^a^	<0.001
Free zinc	19 ± 3^a^	25 ± 6^a^	211 ± 79^b^	20 ± 2^a^	0.003
Protein-bound zinc	8 ± 1^a^	15 ± 5^a^	142 ± 29^b^	12 ± 3^a^	<0.001

Data are given as means ± SE (*n* = 10/group).

Different superscripts indicate significant (*P* < 0.05, Tukey post hoc test) differences.

### Colon microbiota composition, functional capacity, and antibiotic resistance genes

**Fig. 1:**
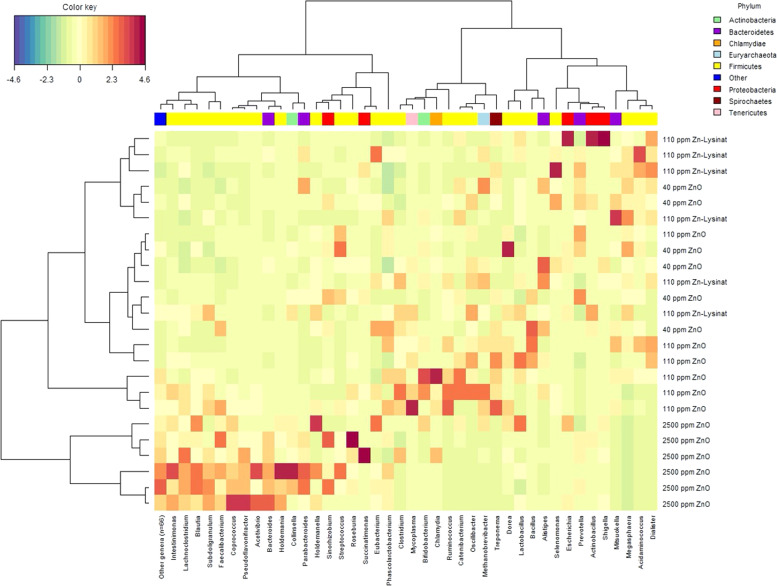
High dietary zinc oxide shapes the microbial community structure in the pig colon at genus level. Heatmap showing the relative abundance of the most prevalent identified bacterial genera and related phyla in colon digesta of piglets (*n* = 6/group) fed diets with added zinc oxide at 40 ppm (40ZnO), 110 ppm (110ZnO), 2500 ppm (2500ZnO), or 110 ppm Zn-Lysinate (110ZnLys).

Colon microbiota composition was determined based on a novel approach using metagenomic sequence alignment to known reference genomes allowing for species-level identification of bacterial taxa. Analysis of relative abundance of the dominant genera (i.e., >0.1% relative abundance in one or more of the experimental groups) revealed only a clear and distinct clustering of colon microbiota in 2500ZnO group as compared with the other groups (Fig. 1). Besides a significant higher abundance of *Bacteroidetes* (i.e., *Bacteroides*, *Parabacteroides*) and *Actinobacteria* (i.e., *Collinsella*), most genera with higher abundance in the 2500ZnO group belonged to the *Firmicutes* phylum (e.g., *Acetivibrio, Blautia, Coprococcus, Faecalibacterium, Subdoligranulum, Holdemania*; Fig. 1, Supplementary Table S5). However, the majority of these genera did not belong to the dominant genera, but were generally of low abundance. In contrast, a significantly lower abundance of the dominating genera *Megasphaera, Dialister, Acidaminococcus*, and *Ruminococcus* was also observed in these animals (Fig. 1, Supplementary Table S5). Besides a subset (*n* = 3) of microbiomes in the 110ZnO group that also formed a separate cluster with a higher abundance of *Bifidobacterium, Ruminococcus*, and *Teponema* and lower abundance of *Megasphaera* and *Prevotella*, no clear patterns between the other groups were observed at phylum and genus level.

**Fig. 2:**
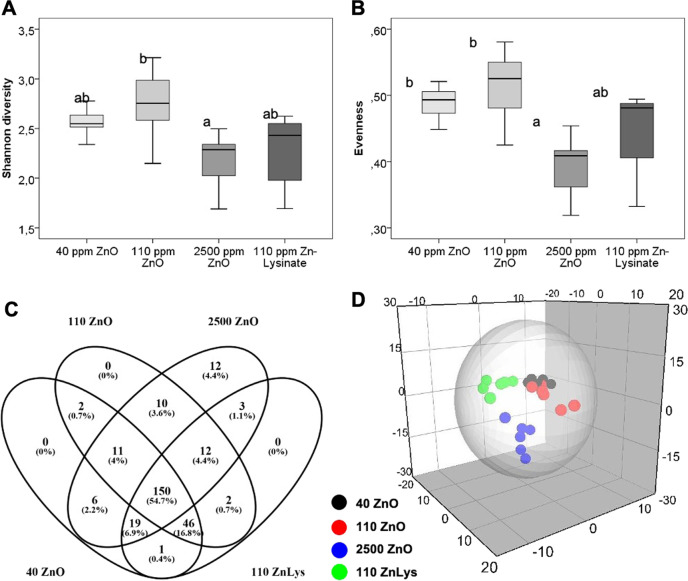
Concentration and chemical form of dietary zinc shape the microbial community structure in the pig colon at species level. Microbial diversity (given as Shannon index H‘, (**a**) and distribution (given as Eveness, **b**), number of shared and unique microbial species (**c**), and PLS-DA scatter plot (**d**) of microbial communities in colon digesta of piglets fed diets with added zinc oxide at 40 ppm (40ZnO), 110 ppm (110ZnO), 2500 ppm (2500ZnO), or 110 ppm Zn-Lysinate (110ZnLys). Different superscripts indicate significant (*P* < 0.05) differences (*n* = 6/group).

At species level, a higher diversity was generally observed in the 110ZnO as compared with the 2500ZnO group, whereas Eveness index was significantly higher in the 40ZnO and 110ZnO group as compared with the 2500ZnO group (Fig. 2a, b). To identify unique and shared bacterial species among groups, a Venn diagram was constructed (Fig. 2), showing that, out of a total of 274 species, 12 species were present only in the 2500ZnO group and another 19 species (*n* = 3, 10, and 6 for 110ZnLys, 110ZnO, and 40ZnO, respectively) were shared between 2500ZnO group and one of the other groups (for details see Supplementary Table S6), whereas no bacterial species were unique to one of the other groups. The majority of species (239, 87.2%) was shared between at least three groups. Finally, the 110ZnLys group had the lowest number of bacterial species shared with only one of the other groups. To further identify the most discriminant bacterial species between groups, PLS-DA was performed and 60 species with highest VIP scores were identified (Fig. 2d, Supplementary Table S7). The PLS-DA revealed unique and distinct patterns for all groups (Fig. 2d). Although the clearest discrimination between groups was due to different abundance or even presence/absence of individual species in 2500ZnO group, other discriminant species were identified in the other groups (Supplementary Table S7).

**Fig. 3:**
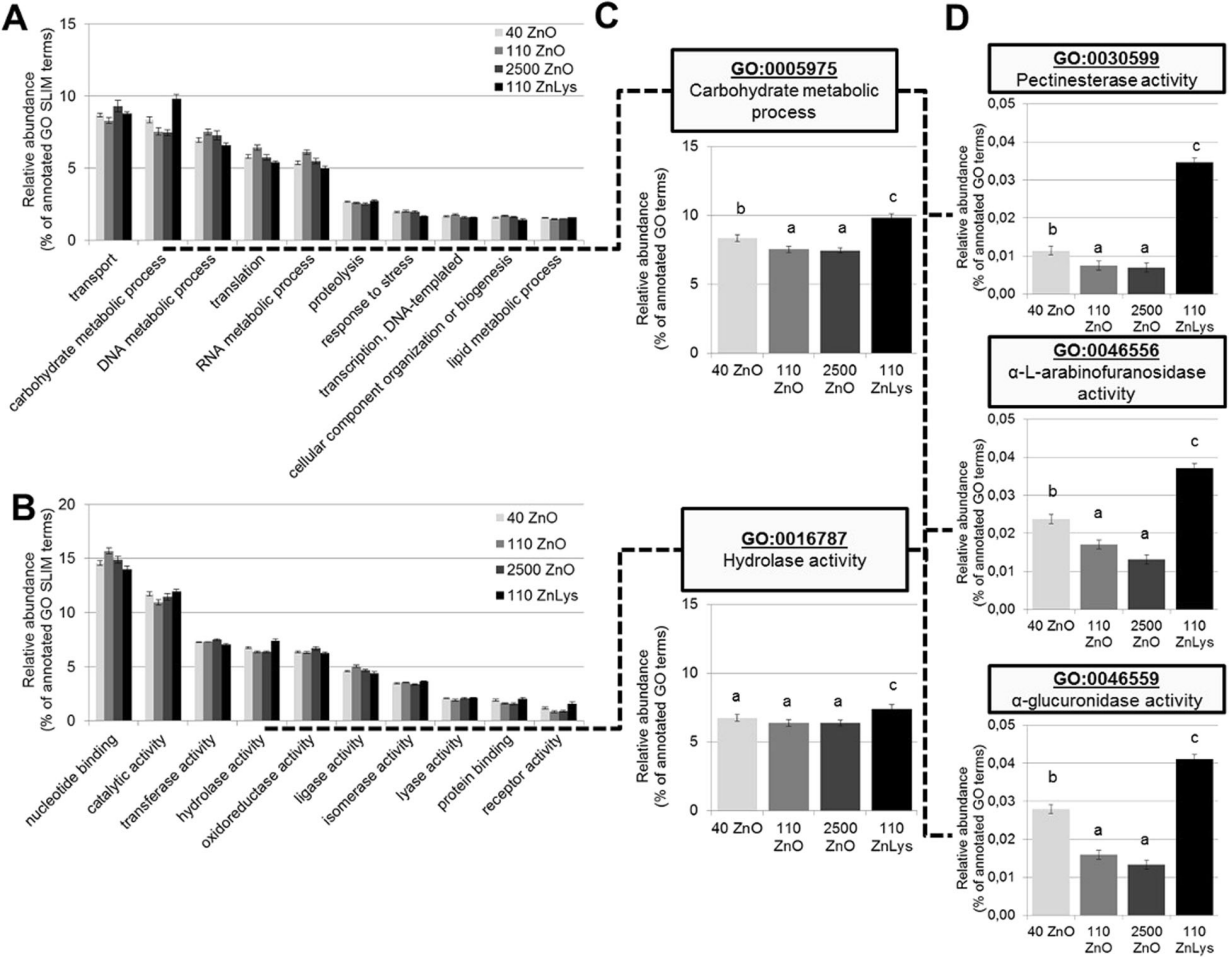
Concentration and chemical form of dietary zinc shape the microbial functional capacity in the pig colon. Relative abundance of significantly affected GO slim terms related to ‘biological process’ (**a**), and ‘molecular function’ (**b**), individual GO slim terms ‘carbohydrate metabolic process’ and ‘hydrolase activity‘ (**c**), as well as individual GO terms ‘pectinesterase activity’, ‘α-L-arabinofuranosidase activity’, and ‘α-glucuronidase activity’ (**d**) obtained from metagenomic sequences of microbial communities in colon digesta of piglets fed diets with added zinc oxide at 40 ppm (40ZnO), 110 ppm (110ZnO), 2500 ppm (2500ZnO), or 110 ppm Zn-Lysinate (110ZnLys). Different superscripts indicate significant (*P* < 0.05) differences (*n* = 6/group).

Analysis of functional capacity based on GO slim terms ‘biological process’ and ‘molecular function’ showed significant differences for a high number of terms involved i.e., in transport, DNA/RNA metabolism, translation and transcription, response to stress, binding processes, and enzyme activity (Fig. 3a, b, Supplementary Tables S8, S9). In contrast to microbiota composition (at the species level), no clear pattern could be determined for a specific dietary group. Of note, a significantly higher abundance of GO slim terms for ‘carbohydrate metabolic process’ (GO:0005975) as well as ‘hydrolase activity’ (GO:0016787) was observed in the 110ZnLys group (Fig. 3c). These two GO slim terms summarize a number of individual enzymes such as ‘pectine esterase activity’ (GO:0030599), ‘α-L-arabinofuranosidase activity’ (GO:0046556), or ‘α-glucuronidase activity’ (GO:0046559), which all showed a significantly higher abundance in both the 40ZnO as well as the 110ZnLys groups as compared with 110ZnO and 2500ZnO groups, respectively (Fig. 3d). In contrast, individual GO terms ‘metal ion binding’ (GO:0046872), ‘metal ion transport’(GO:0030001), penicillin binding’ (GO: 0008658), and ‘β-lactam catabolism’ (GO:0030655) had the highest abundance in 2500ZnO group and the lowest levels in the 110ZnLys group (Supplementary Fig. S1).

Analysis of total and individual SCFA revealed striking differences between dietary groups (Table 2). Lowest (*P* < 0.05) levels for total SCFA were determined in the 2500ZnO group followed by the 111ZnO group and with 40ZnO as well as 110ZnLys groups both having highest levels. A similar pattern was observed for propionate as well as n-butyrate levels, which had the highest total levels and molar ratio in the 40ZnO and 110ZnLys groups. Total acetate was lower in the 2500ZnO group but did not differ between the other groups resulting in a shift towards a higher acetate molar ratio in both the 2500ZnO and 110ZnO group as compared with the 40ZnO and 110ZnLys groups, respectively. The NH_4_ level was lower (*P* < 0.05) in the 2500ZnO group as compared with the other groups. No effects were observed for BCFA. Total and individual d- or l-lactate was only detected in small amounts in the colon and did not differ between groups.

**Table 2:** Bacterial metabolite concentrations in colon digesta of piglets fed diets with added zinc oxide at 40 ppm (40ZnO), 110 ppm (110ZnO), 2500 ppm (2500ZnO), or 110 ppm Zn-Lysinate (110ZnLys) over a period of 3 weeks.

Item	40ZnO	110ZnO	2500ZnO	110ZnLys	*P* value
mmol/l					
d-Lactate	0.2 ± 0.1	0.2 ± 0.1	0.1 ± 0.0	0.1 ± 0.0	0.347
l-Lactate	0.2 ± 0.1	0.2 ± 0.1	0.1 ± 0.0	0.1 ± 0.0	0.279
Total lactate	0.3 ± 0.2	0.4 ± 0.2	0.1 ± 0.0	0.1 ± 0.0	0.343
Total SCFA	81.5 ± 2.7^c^	68.4 ± 4.3^b^	45.9 ± 2.8^a^	80.0 ± 3.7^c^	<0.001
Acetate	41.3 ± 1.6^b^	38.3 ± 2.6^b^	27.1 ± 1.6^a^	40.6 ± 2.4^b^	<0.001
Propionate	22.7 ± 0.9^c^	17.1 ± 1.1^b^	12.1 ± 0.9^a^	23.1 ± 1.4^c^	<0.001
n-Butyrate	11.3 ± 0.9^c^	8.3 ± 0.8^b^	5.0 ± 0.3^a^	10.7 ± 0.7^c^	<0.001
BCFA	2.6 ± 0.6	2.8 ± 0.3	1.2 ± 0.2	2.2 ± 0.4	0.056
NH_4_	10.6 ± 1.2^b^	11.4 ± 1.0^b^	4.6 ± 0.9^a^	11.6 ± 1.3^b^	<0.001
Acetate (Mol %)	50.8 ± 1.6^a^	55.8 ± 1.2^b^	59.0 ± 0.9^b^	50.6 ± 1.0^a^	<0.001
Propionate (Mol %)	27.9 ± 0.8^b^	25.0 ± 0.6^a^	26.2 ± 0.7^ab^	28.8 ± 1.0^c^	0.012
n-Butyrate (Mol %)	13.8 ± 0.5^b^	12.1 ± 0.9^ab^	10.0 ± 0.3^a^	13.3 ± 0.3^b^	0.046

Data are given as means ± SE (*n* = 10/group).

Different superscripts indicate significant (*P* < 0.05, Tukey post hoc test) differences.

**Fig. 4:**
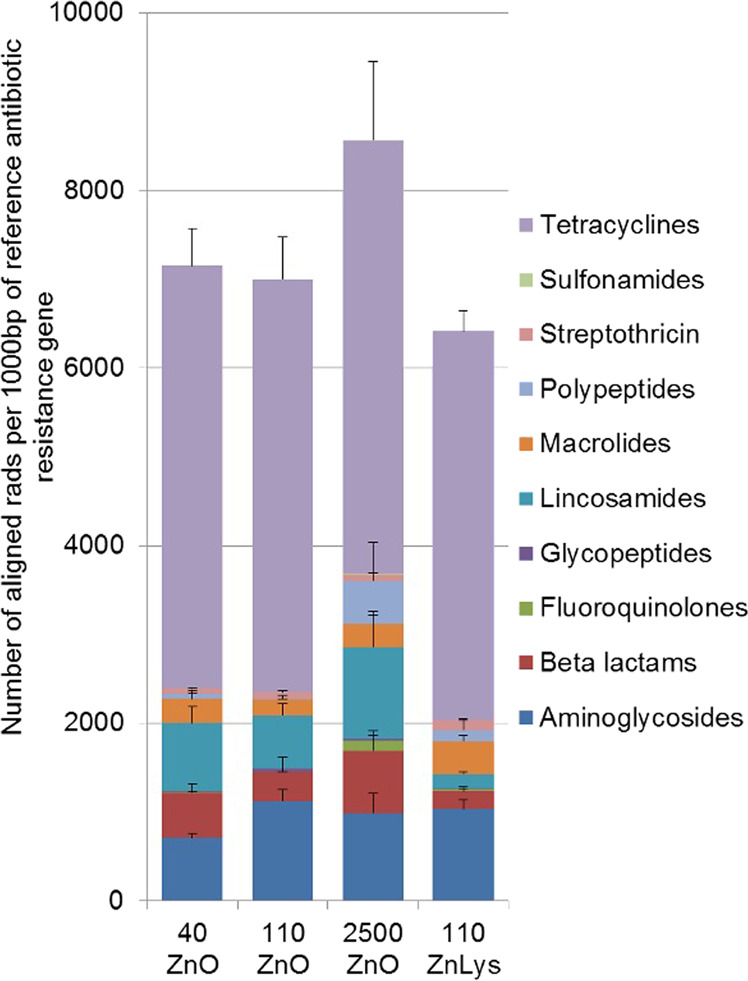
Concentration and chemical form of dietary zinc alter the antibiotic resistance gene abundance in the pig colon. Relative abundance of antibiotic resistance genes (given as number of aligned reads per 1000 bp of reference gene) identified in colon microbiomes of piglets (*n* = 6/group) fed diets with added zinc oxide at 40 ppm (40ZnO), 110 ppm (110ZnO), 2500 ppm (2500ZnO), or 110 ppm Zn-Lysinate (110ZnLys) over a period of 3 weeks. Individual gene abundance within each class of antibiotics is given in Supplementary Table S10.

Finally, the relative abundance of known AR genes revealed no clear differences between groups regarding the total number of AR genes and for genes conferring resistance against a certain antimicrobial class. (Fig. 4). Individual genes showed a high variability in their abundance and in relation to dietary zinc (Supplementary Table 10). However, individual genes (*aph(3”)-Ib, bla*_*ROB*_, *pat(A), lnu(C), arnA*) showed a higher abundance in the 2500ZnO group, whereas *cfxA2*, and *erm(G)* had a higher abundance in the 40ZnO and 110ZnLys groups.

## Discussion

High levels of dietary ZnO have been and are still used as alternatives to antibiotic growth promoters in pigs [4, 5]. One major effect of very high amounts of ZnO in pig nutrition is the modulation of the intestinal microbial community composition, [8, 14, 22, 23]. However, less is yet known about the influence of lower concentrations of inorganic and organic zinc sources. In the present study, measurements regarding homeostatic regulation of zinc uptake, organ zinc load and excretion as well as zinc fractions in the GIT were performed to obtain a better view whether this could be associated to intestinal microbial responses. In fact, organ zinc status was clearly affected only by the high ZnO diet as revealed by higher zinc concentration in several organs, different expression of small intestinal zinc transporters (higher expression of exporter *ZnT1*, lower expression of zinc importer *ZIP4*), and a higher expression of metallothioneins *MT1A* and *MT2B*, which is a good indicator for homeostatic regulation [24, 25]. The trend towards lower apparent ileal zinc digestibility in the high ZnO group reflects homeostatic counter-regulation or a limitation of absorptive capacity. In contrast, there was no clear distinction for these parameters between the other dietary groups, which was also reflected by similar small intestinal luminal concentrations of total, protein-bound, and free zinc fractions. The data suggest that only minor differences existed between the 40ZnO, 110ZnO, and 110ZnLys, supporting previous findings [12, 26, 27]. However, the study was not designed to evaluate the bioavailability of ZnO versus ZnLys for both, the animal as well as intestinal microbiota. Especially the protein-bound zinc fraction could be composed of many different compounds including Zn-amino acid chelates or more complex molecules, where Zn is bound to oligo- or polypeptides. It is not clear whether organic zinc compounds might act differently on intestinal bacterial communities as compared with ZnO or free zinc ions. A previous study has shown the fraction of free zinc ions, but also protein-bound zinc, correlated with various and partially different parameters of intestinal microbial ecology in the colon of pigs fed high dietary ZnO levels [14]. The major effect is likely exerted by bacteriostatic or toxic effects of zinc ions in the gut lumen [14, 28]. However, conclusions on genus level might be misleading towards a general susceptibility or tolerance of all members of a certain genus against zinc. For example, some members of the same genus (e.g. *Bacteroides, Bifidobacterium, Clostridium*) have been shown to exert a higher tolerance, whereas others were more susceptible against zinc in vitro [28]. In order to analyze the influence of zinc source and chemical form beyond genus level and omitting possible bias by analysis of 16S rRNA gene amplicons, we used a novel approach allowing to identify bacterial species by alignment of metagenomic reads to known taxa [19]. Further downstream analysis and multivariate analysis was used to identify those taxa that distinguished between the feeding groups. Of note, a previous study has postulated that high ZnO may increase *Methanobrevibacter* spp. and thereby metabolic pathways for increased methanogenesis [23]. However, in this study microbial metabolic function was predicted based on 16S rRNA gene amplicons. Here, we could not find differences for the *Methanobrevibacter* genus. However, a trend (*P* < 0.1) for a higher abundance of the GO term ‘methanogenesis’ (GO:0015948, Table S8) was observed in the 2500ZnO group, and lowest with the 110ZnLys group, which could point in the same direction as previously observed [23]. It is also possible that other hydrogen sinks played a role in pigs fed the 110ZnLys diets, because hydrogenotrophic microorganisms generally belong to three different groups, the sulfate-reducing bacteria, methanogens and acetogens [29]. As an indicator, a typical sulfate-reducing species, *Desulfovibrio piger*, was found at higher abundance in the 110ZnLys animals. *D. piger*, is likely involved in sulfate reduction through complex cross-feeding processes with *Bacteroides* and *Actinobacterium* [30]. Measurements of methanogenesis or formation of hydrogen sulfide in the context of metabolic cross-feeding could help to further evaluate this finding in the future. Species-level analysis not only revealed differences for high dietary ZnO levels (as already visible at genus level), but furthermore showed that the 110ZnLys group clustered separately from the other treatments. Reasons are yet not clear, because, as indicated above, no clear differences in zinc homeostasis or intestinal zinc fractions were found compared with the 110ZnO group. This could be due to host-driven selection of certain bacterial taxa because even small differences in dietary zinc supply have been previously shown to significantly alter systemic and gut-associated immune reactions in growing pigs [31, 32]. It could be also speculated that certain fractions within protein-bound zinc have a specific effect on bacterial cells or, in turn, did not exert inhibitory effects as compared with diets containing 110 or 2500 mg zinc/kg from ZnO. During a feeding period of 3 weeks, this could contribute to a forward selection of microbial communities being more metabolically active with ZnLys as compared with ZnO. In this context, the higher abundance of GO slim terms for ‘carbohydrate metabolic process’ (GO:0005975) as well as ‘hydrolase activity’ (GO:0016787) in the 110ZnLys group as well as the 40ZnO and their individual child-terms such as ‘pectinesterase activity’ (GO:0030599), ‘α-L-arabinofuranosidase activity’ (GO:0046556), or ‘α-glucuronidase activity’ (GO:0046559) may support the latter hypothesis. They indicate a selection towards increased glycan foraging in these groups. Increased glycan foraging could be a reason for the above-mentioned effects on hydrogen cross-feeding. In addition, one interesting finding was a very high abundance of *Dialister succinatiphilus* being able to decarboxylate succinate to propionate among pigs fed the 110ZnLys diets. Because shifts in intestinal microbiota communities in the present study were determined based on genomic DNA, we also measured the concentration of bacterial metabolites allowing to better distinguishing between the presence/absence of species and their genes and their actual metabolic activity. Supporting possible cross-feeding mechanisms, the highest concentration of bacterial metabolites was found in the 40ZnO and the 110ZnLys groups. Also, the effects of high dietary ZnO level on bacterial metabolite concentration in the large intestine are in line with previous findings [14, 16, 22]. Interestingly, lower total SCFA and NH_4_ were in part also observed with the 110ZnO group supporting possible dose-dependent effects [16]. Whether this is due to true bacteriostatic effects of zinc ions or e.g., differences in metabolite absorption by the host is yet not clear and may warrant further studies. One previous observation was an increased concentration of lactate in the colon of pigs fed high dietary ZnO [22]. This was explained by the authors with differences in the availability of fermentable substrates due to feeding time and nutrient flow to the large intestine. In the present study, all pigs received the last meal 4 h before euthanasia for sampling. To exclude that the difference in bacterial metabolic activity regarding SCFA and NH_4_ could be due to other factors affecting the fractional nutrient flow to the large intestine, we determined the ileal nutrient digestibility but did not observe significant differences between groups. Thus, the observed differences in bacterial metabolic activity between the groups were most likely due to bacteriostatic effects of zinc ions (or other binding forms) in the gut lumen. Supporting this hypothesis, individual GO terms ‘metal ion binding’ (GO:0046872), ‘metal ion transport’(GO:0030001), penicillin binding’ (GO: 0008658), and ‘β-lactam catabolism’ (GO:0030655) had the highest abundance in 2500ZnO group and the lowest levels in the 110ZnLys group. This would support a forward selection of bacterial taxa with specific evolutionary adaptations through dietary zinc amount and source. It has been hypothesized already decades ago that high ZnO diets for weaned piglets could promote zinc tolerance and concomitant antibiotic resistance in intestinal bacteria and in manure [33–35]. For example, the use of high levels of ZnO in swine diets has been lately attributed with an increased prevalence of intestinal multi-resistant *Escherichia coli* [10, 11]. In the present study, metagenomic reads were aligned to known AR genes using the CARD database to obtain a general overview of the gut resistome in relation to zinc in the diet. The data show that, despite a generally higher abundance of cumulative AR genes in the 2500ZnO group, significant diets effects were only observed for a few individual genes (i.e., *aph(3”)-Ib, bla*_*ROB*_, *pat(A), lnu(C), arnA*) and not AR genes in general. Genes such as *aph(3”)-Ib*, *bla*_*ROB*_, and *arnA* can be commonly found in enterobacteria [36]. In addition, *aph(3”)-Ib*, for example, has been recently shown to be associated with higher zinc tolerance in intestinal *E. coli* from weaned piglets [37]. This is supported by our findings here and by previous studies showing an increased abundance and diversity of enterobacteria with high dietary zinc levels in pigs [16, 22, 38]. The fact that no clear difference between the other feeding groups were observed suggests that the organic zinc source and ZnO levels did not contribute to increased abundance of intestinal AR genes in general. Assuming that the intestinal microbiota composition stabilizes 2 weeks after the weaning in piglets, a possible forward selection of bacterial taxa by high ZnO as the main driver for AR gene abundance would not lead to significant changes in AR gene abundance beyond the 3-week period studied here. However, the present study does not allow drawing conclusions on increased resistance acquisition or possible co-selection among certain members of the microbial communities. Increased horizontal gene transfer due to high zinc concentrations (i.e. chronic exposure) might thus be associated with increased AR gene abundance beyond this time frame. However, this has yet not been shown in pigs in vivo and requires further elucidation.

## Conclusions

The current study confirms the strong influence of high dietary zinc concentrations on zinc homeostasis, the large intestinal microbiome and partly also on AR genes. Further it reveals that Zn-Lysinate as organic zinc source favors a differentially composed microbiome, enriched in genes that may promote glycan foraging and maintain a desired formation of beneficial metabolites. Consistent with previous findings, there was evidence of a weak but detectable increase in AR genes associated with high levels of ZnO.

## Supplementary information

Supplemental Table S1

Supplemental Table S2

Supplemental Table S3

Supplemental Table S4

Supplemental Table S5

Supplemental Table S6

Supplemental Table S7

Supplemental Table S8

Supplemental Table S9

Supplemental Table S10

Supplemental Figure S1
