# Supplementary material for: Concentration and chemical form of dietary zinc shape the porcine colon microbiome, its functional capacity and antibiotic resistance gene repertoire
Source: ISME J. 2020 Aug 3;14(11):2783–93. doi: 10.1038/s41396-020-0730-3 (PMC7784847; doi:10.1038/s41396-020-0730-3)
Supplement: Supplementary file 1 — Supplemental Table S1 [file 41396_2020_730_MOESM1_ESM.docx]

Supplemental Table S1. Ingredients and analyzed chemical composition of experimental diets.

|  | Dietary treatments | | | |
| --- | --- | --- | --- | --- |
|  | 40 ZnO | 110 ZnO | 2500 ZnO | 110 ZnLys |
| *Ingredients* | *g/kg as fed* | | | |
| Wheat | 298.1 | 298.1 | 297.6 | 299.6 |
| Corn | 120.6 | 120.6 | 120.6 | 120.6 |
| Barley | 220.0 | 220.0 | 220.0 | 220.0 |
| Soybean meal (CP:48%) | 190.0 | 190.0 | 190.0 | 190.0 |
| Skimmed milk powder | 110.0 | 110.0 | 110.0 | 110.0 |
| Limestone | 14.5 | 14.5 | 14.5 | 14.5 |
| Soy oil | 13.7 | 13.7 | 13.7 | 13.7 |
| Mineral/Vitamin premix ^1)^ | 12.0 | 12.0 | 12.0 | 12.0 |
| Monocalciumphosphate | 10.2 | 10.2 | 10.2 | 10.2 |
| L-Lysine | 3.10 | 3.10 | 3.10 | 3.00 |
| DL Methionine | 1.38 | 1.38 | 1.38 | 1.38 |
| L-Threonine | 1.19 | 1.19 | 1.19 | 1.19 |
| L-Tryptophane | 0.38 | 0.38 | 0.38 | 0.38 |
| Iron-(II)-sulphate | 0.19 | 0.19 | 0.19 | 0.19 |
| Copper-(II)-sulphate | 0.19 | 0.19 | 0.19 | 0.19 |
| Manganese-(II)-sulphate | 0.13 | 0.13 | 0.13 | 0.13 |
| Corn starch | 2.8 | 2.7 | - | 2.0 |
| Zinc oxide | 0.05 | 0.15 | 3.38 | - |
| Zink-Lysinate | - | - | - | 0.88 |
| *Analyzed composition* |  | | | |
| ME ^2)^ (MJ/kg) | 14.1 | 14.1 | 14.1 | 14.1 |
|  | *g/kg dry matter* | | | |
| Dry matter | 906 | 905 | 907 | 908 |
| Ash | 59 | 66 | 65 | 60 |
| Crude protein | 206 | 208 | 209 | 213 |
| Ether extract | 26 | 30 | 28 | 25 |
| Crude fiber | 23 | 24 | 22 | 23 |
| Starch | 380 | 397 | 374 | 380 |
| Phosphorus | 6.9 | 6.7 | 6.6 | 6.4 |
| Calcium | 10.2 | 9.8 | 9.4 | 9.3 |
| Potassium | 8.4 | 8.2 | 8.0 | 7.9 |
| Sodium | 2.3 | 2.1 | 2.0 | 1.9 |
| Magnesium | 2.0 | 2.0 | 2.0 | 1.9 |
|  | *mg/kg dry matter* | | | |
| Iron | 203 | 208 | 216 | 181 |
| Zinc | 94 | 149 | 2183 | 136 |
| Copper | 20 | 19 | 20 | 17 |
| Manganese | 48 | 47 | 49 | 45 |

^1)^ Contents per kg Premix : 400000 I.U. vit. A (acetate) ; 120000 I.U. vit. D3 ; 8000 mg vit. E (α-tocopherole acetate) ; 200 mg vit. K3 (MSB) ; 250 mg vit. B1 (mononitrate) ; 420 mg vit. B2 (cryst. Riboflavin) ; 2500 mg niacin (niacinamide) ; 400 mg Vit. B6 (Hcl) ; 2000 µg vit. B12 ; 25000 µg Biotin (commercial, feed grade) ; 1000 mg pantothenic acid (Ca d-pantothenate) ; 100 mg folic acid (cryst. Commercial feed grade) ; 80000 mg choline (chloride) ; 20 mg Se (Na-selenite) ; 45 mg J (Ca-iodate) ; 130 g Na (NaCl) ; 55 g Mg (sulfate)

^2)^ Calculated according to GfE (2008)
