# Supplementary material for: Concentration and chemical form of dietary zinc shape the porcine colon microbiome, its functional capacity and antibiotic resistance gene repertoire
Source: ISME J. 2020 Aug 3;14(11):2783–93. doi: 10.1038/s41396-020-0730-3 (PMC7784847; doi:10.1038/s41396-020-0730-3)
Supplement: Supplementary file 2 — Supplemental Table S2 [file 41396_2020_730_MOESM2_ESM.docx]

**Supplemental Table S2**. Ileal apparent nutrient digestibility in piglets fed diets with added zinc oxide at 40 ppm (40 ZnO), 110 ppm (110 ZnO), 2500 ppm (2500ZnO), or 110 ppm Zn-Lysinate (110ZnLys) over a period of three weeks (n= 10/group).

|  | 40 ZnO | 110 ZnO | 2500 ZnO | 110 ZnLys | P-Value |
| --- | --- | --- | --- | --- | --- |
| *Ileal digestibility* | *(%)* | | | |  |
| Organic matter | 78.6 ± 1.3 | 77.5 ± 1.9 | 75.5 ± 1.0 | 76.4 ± 1.0 | 0.435 |
| Crude protein | 82.3 ± 1.7 | 83.2 ± 1.5 | 80.8 ± 2.2 | 82.5 ± 1.1 | 0.787 |
| Ether extract | 94.6 ± 1.3 | 96.3 ± 0.7 | 93.8 ± 1.2 | 93.8 ± 0.9 | 0.351 |
| Starch | 97.7 ± 0.4 | 97.2 ± 0.5 | 96.1 ± 0.6 | 96.6 ± 0.5 | 0.157 |
| Zinc | 15.5 ± 5.5 | 9.6 ± 5.3 | 2.4 ± 9.1 | 14.4 ± 5.2 | 0.475 |
