# Supplementary material for: Concentration and chemical form of dietary zinc shape the porcine colon microbiome, its functional capacity and antibiotic resistance gene repertoire
Source: ISME J. 2020 Aug 3;14(11):2783–93. doi: 10.1038/s41396-020-0730-3 (PMC7784847; doi:10.1038/s41396-020-0730-3)
Supplement: Supplementary file 3 — Supplemental Table S3 [file 41396_2020_730_MOESM3_ESM.docx]

**Supplemental Table S3**. Organ zinc concentration in piglets fed diets with added zinc oxide at 40 ppm (40 ZnO), 110 ppm (110 ZnO), 2500 ppm (2500ZnO), or 110 ppm Zn-Lysinate (110ZnLys) over a period of three weeks. Different superscripts indicate significant (P<0.05) differences (n= 10/group).

|  | 40 ZnO | | 110 ZnO | | 2500 ZnO | 110 ZnLys | P-Value |
| --- | --- | --- | --- | --- | --- | --- | --- |
| *Organ* | mg/kg DM | | | | | |  |
| Jejunum | 80.7 ± 2.6^a^ | 80.3 ± 1.6^a^ | | 659.5 ± 73.1^b^ | | 87.0 ± 3.6^a^ | <0.001 |
| Metacarpal IV | 84.4 ± 2.2^a^ | 106.5 ± 4.3^b^ | | 233.8 ± 11.2^c^ | | 99.1 ± 2.9^ab^ | <0.001 |
| Liver | 129.2 ± 5.3^a^ | 180.6 ± 11.0^a^ | | 1504.7 ± 88.8^b^ | | 183.6 ± 20.8^a^ | <0.001 |
| Kidney | 99.7 ± 4.9^a^ | 101.1 ± 3.8^a^ | | 412.7 ± 27.7^b^ | | 97.3 ± 1.7^a^ | <0.001 |
| Pancreas | 119.2 ± 8.6^a^ | 124.2 ± 7.6^a^ | | 1532.5 ± 151.9^b^ | | 118.8 ± 7.4^a^ | <0.001 |
