# Supplementary material for: Concentration and chemical form of dietary zinc shape the porcine colon microbiome, its functional capacity and antibiotic resistance gene repertoire
Source: ISME J. 2020 Aug 3;14(11):2783–93. doi: 10.1038/s41396-020-0730-3 (PMC7784847; doi:10.1038/s41396-020-0730-3)
Supplement: Supplementary file 4 — Supplemental Table S4 [file 41396_2020_730_MOESM4_ESM.docx]

**Supplemental Table S4**. Relative expression of zinc transporters (*ZIP4, Znt1*) and metallothionein (*MT-1, MT-2b*) in jejunal tissue of piglets fed diets with added zinc oxide at 40 ppm (40 ZnO), 110 ppm (110 ZnO), 2500 ppm (2500ZnO), or 110 ppm Zn-Lysinate (110ZnLys) over a period of three weeks. Different superscripts indicate significant (P<0.05) differences (n= 6/group).

|  | 40 ZnO | | 110 ZnO | | 2500 ZnO | 110 ZnLys | P-Value |
| --- | --- | --- | --- | --- | --- | --- | --- |
| *Gene name* | relative Expression | | | | | |  |
| *ZIP4* | 1.00 ± 0.06^b^ | 1.19 ± 0.09^b^ | | 0.16 ± 0.01^a^ | | 1.10 ± 0.09^b^ | <0.001 |
| *ZnT1* | 1.00 ± 0.06^a^ | 1.70 ± 0.08^b^ | | 2.56 ± 0.22^c^ | | 1.39 ± 0.14^ab^ | <0.001 |
| *MT1A* | 1.18 ± 0.21^a^ | 1.66 ± 0.72^a^ | | 123.00 ± 19.24^b^ | | 2.11 ± 0.44^a^ | <0.001 |
| *MT2B* | 1.07 ± 0.15^a^ | 1.01 ± 0.39^a^ | | 34.51 ± 6.40^b^ | | 1.74 ± 0.35^a^ | <0.001 |
