# Supplementary material for: Concentration and chemical form of dietary zinc shape the porcine colon microbiome, its functional capacity and antibiotic resistance gene repertoire
Source: ISME J. 2020 Aug 3;14(11):2783–93. doi: 10.1038/s41396-020-0730-3 (PMC7784847; doi:10.1038/s41396-020-0730-3)
Supplement: Supplementary file 5 — Supplemental Table S5 [file 41396_2020_730_MOESM5_ESM.docx]

**Supplemental Table S5**. Relative abundance of bacterial genera in colon digesta of piglets fed diets with added zinc oxide at 40 ppm (40 ZnO), 110 ppm (110 ZnO), 2500 ppm (2500ZnO), or 110 ppm Zn-Lysinate (110ZnLys) over a period of three weeks. Different superscripts indicate significant (P<0.05) differences (n= 6/group).

|  | 40 ZnO | 110 ZnO | 2500 ZnO | 110 ZnLys | P-Value |
| --- | --- | --- | --- | --- | --- |
| *Genus* | % of aligned sequences | | | |  |
| *Prevotella* | 33.91 ± 6.76 | 17.05 ± 7.47 | 18.90 ± 4.69 | 17.91 ± 6.64 | 0.237 |
| *Lactobacillus* | 7.77 ± 1.81 | 13.24 ± 5.94 | 13.22 ± 5.87 | 14.82 ± 2.73 | 0.704 |
| *Megasphaera* | 11.39 ± 2.58^b^ | 6.72 ± 1.82^ab^ | 0.01 ± 0.01^a^ | 14.68 ± 2.50^b^ | <0.001 |
| *Phascolarctobacterium* | 6.36 ± 2.32 | 9.98 ± 2.12 | 8.40 ± 1.50 | 7.10 ± 1.86 | 0.594 |
| *Clostridium* | 4.11 ± 1.19 | 9.98 ± 2.21 | 4.68 ± 1.86 | 5.87 ± 2.25 | 0.166 |
| *Alistipes* | 8.44 ± 3.88 | 3.00 ± 1.41 | 0.30 ± 0.11 | 4.28 ± 2.69 | 0.164 |
| *Dialister* | 1.73 ± 0.83^ab^ | 3.30 ± 1.45^ab^ | 0.04 ± 0.03^a^ | 5.58 ± 1.45^b^ | 0.014 |
| *Roseburia* | 1.79 ± 0.27 | 2.34 ± 0.75 | 5.39 ± 2.58 | 0.99 ± 0.16 | 0.144 |
| *Ruminococcus* | 1.49 ± 0.33^a^ | 5.40 ± 1.77^b^ | 0.43 ± 0.05^a^ | 1.62 ± 0.65^ab^ | 0.008 |
| *Bacteroides* | 1.31 ± 0.17^a^ | 0.88 ± 0.17^a^ | 4.98 ± 1.07^b^ | 0.68 ± 0.24^a^ | <0.001 |
| *Mitsuokella* | 2.00 ± 0.60 | 2.10 ± 0.77 | 0.02 ± 0.02 | 3.43 ± 1.74 | 0.150 |
| *Oscillibacter* | 1.40 ± 0.37 | 2.31 ± 0.55 | 0.54 ± 0.06 | 1.40 ± 0.58 | 0.073 |
| *Eubacterium* | 0.81 ± 0.33 | 0.58 ± 0.20 | 1.23 ± 0.42 | 1.20 ± 0.43 | 0.517 |
| *Selenomonas* | 0.97 ± 0.52 | 0.27 ± 0.08 | 0.01 ± 0.00 | 1.58 ± 1.07 | 0.271 |
| *Succinatimonas* | 0.24 ± 0.12 | 0.07 ± 0.06 | 1.35 ± 1.35 | 0.28 ± 0.24 | 0.552 |
| *Dorea* | 0.53 ± 0.36 | 0.62 ± 0.12 | 0.38 ± 0.11 | 0.26 ± 0.10 | 0.619 |
| *Bacillus* | 0.76 ± 0.50 | 0.86 ± 0.47 | n.d. | 0.002 ± 0.002 | 0.172 |
| *Streptococcus* | 0.57 ± 0.30 | 0.33 ± 0.22 | 0.51 ± 0.30 | 0.10 ± 0.04 | 0.502 |
| *Mycoplasma* | 0.21 ± 0.11 | 0.88 ± 0.45 | n.d. | 0.18 ± 0.16 | 0.086 |
| *Bifidobacterium* | 0.15 ± 0.06 | 0.59 ± 0.27 | 0.15 ± 0.10 | 0.15 ± 0.08 | 0.144 |
| *Treponema* | 0.28 ± 0.07^ab^ | 0.60 ± 0.17^b^ | n.d. | 0.15 ± 0.08^a^ | 0.003 |
| *Escherichia* | 0.14 ± 0.08 | n.d. | 0.58 ± 0.47 | 0.28 ± 0.19 | 0.440 |
| *Acidaminococcus* | 0.28 ± 0.03^ab^ | 0.21 ± 0.08^ab^ | 0.003 ± 0.003^a^ | 0.42 ± 0.16^b^ | 0.031 |
| *Faecalibacterium* | 0.15 ± 0.07^a^ | 0.19 ± 0.07^ab^ | 0.40 ± 0.07^b^ | 0.11 ± 0.02^a^ | 0.017 |
| *Coprococcus* | 0.05 ± 0.01^a^ | 0.10 ± 0.03^a^ | 0.64 ± 0.16^b^ | 0.04 ± 0.01^a^ | <0.001 |
| *Shigella* | 0.11 ± 0.10 | n.d. | 0.58 ± 0.55 | 0.11 ± 0.07 | 0.480 |
| *Blautia* | 0.09 ± 0.02^a^ | 0.13 ± 0.03^a^ | 0.45 ± 0.09^b^ | 0.10 ± 0.03^a^ | <0.001 |
| *Acetivibrio* | 0.08 ± 0.01^a^ | 0.11 ± 0.03^ab^ | 0.49 ± 0.19^b^ | 0.08 ± 0.01^a^ | 0.019 |
| *Methanobrevibacter* | 0.10 ± 0.07 | 0.19 ± 0.08 | 0.06 ± 0.03 | 0.12 ± 0.05 | 0.502 |
| *Collinsella* | 0.07 ± 0.01^ab^ | 0.05 ± 0.01^a^ | 0.27 ± 0.10^b^ | 0.09 ± 0.03^ab^ | 0.039 |
| *Parabacteroides* | 0.09 ± 0.06^ab^ | 0.02 ± 0.01^a^ | 0.26 ± 0.09^b^ | 0.05 ± 0.04^ab^ | 0.041 |
| *Intestinimonas* | 0.04 ± 0.01^a^ | 0.09 ± 0.02^ab^ | 0.21 ± 0.06^b^ | 0.04 ± 0.01^a^ | 0.003 |
| *Catenibacterium* | 0.08 ± 0.02 | 0.17 ± 0.07 | 0.04 ± 0.02 | 0.07 ± 0.04 | 0.181 |
| *Subdoligranulum* | 0.04 ± 0.01^a^ | 0.08 ± 0.02^ab^ | 0.14 ± 0.03^b^ | 0.07 ± 0.02^ab^ | 0.027 |
| *Actinobacillus* | 0.05 ± 0.04 | 0.003 ± 0.003 | 0.03 ± 0.02 | 0.21 ± 0.14 | 0.207 |
| *Holdemania* | n.d. | 0.007 ± 0.003^a^ | 0.25 ± 0.10^b^ | n.d. | 0.005 |
| *Lachnoclostridium* | 0.03 ± 0.01^a^ | 0.05 ± 0.01^a^ | 0.12 ± 0.02^b^ | 0.04 ± 0.01^a^ | <0.001 |
| *Holdemanella* | 0.04 ± 0.01 | 0.04 ± 0.01 | 0.11 ± 0.05 | 0.04 ± 0.02 | 0.226 |
| *Pseudoflavonifractor* | 0.01 ± 0.01^a^ | 0.02 ± 0.01^a^ | 0.16 ± 0.04^b^ | 0.02 ± 0.01^a^ | <0.001 |
| *Chlamydia* | 0.01 ± 0.01 | 0.15 ± 0.09 | 0.04 ± 0.04 | 0.003 ± 0.003 | 0.168 |
| *Sinorhizobium* | 0.06 ± 0.02 | 0.02 ± 0.01 | 0.10 ± 0.04 | 0.09 ± 0.00 | 0.079 |
| Other genera (n=66) | 0.33 ± 0.05^a^ | 0.43 ± 0.13^a^ | 1.09 ± 0.16^b^ | 0.25 ± 0.08^a^ | <0.001 |

n.d. - not detected
