# Supplementary material for: Concentration and chemical form of dietary zinc shape the porcine colon microbiome, its functional capacity and antibiotic resistance gene repertoire
Source: ISME J. 2020 Aug 3;14(11):2783–93. doi: 10.1038/s41396-020-0730-3 (PMC7784847; doi:10.1038/s41396-020-0730-3)
Supplement: Supplementary file 6 — Supplemental Table S6 [file 41396_2020_730_MOESM6_ESM.docx]

**Supplemental Table S6**. Unique and shared bacterial species between the experimental groups, excluding the 150 species being shared among all groups, respectively.

| **Group** | **Unique species** |
| --- | --- |
| 40 ZnO | - |
| 110 ZnO | - |
| 2500 ZnO | *Bacteroides barnesiae,*  *Alistipes senegalensis*  *unclassified Clostridium sp. 48*  *Bacteroides uniformis CAG:3*  *Ruminococcus gnavus*  *Prevotella dentasini*  *Marvinbryantia formatexigens*  *Bacteroides acidifaciens*  *unclassified Blautia sp. 1*  *Bacteroides gallinarum*  *unclassified Prevotella sp. 1*  *unclassified Prevotella sp. 4* |
| 110 ZnLys | - |
| 40 ZnO  110 ZnO | *unclassified Clostridium sp. 13*  *Parabacteroides merdae* |
| 40 ZnO  2500 ZnO | *unclassified Prevotella sp. 7*  *unclassified Bacteroides sp. 1*  *unclassified Tannerella sp.*  *Bacteroides thetaiotaomicron*  *unclassified Parabacteroides sp. 2*  *Alloprevotella tannerae* |
| 40 ZnO  110 ZnLys | *unclassified Roseburia sp. 6* |
| 110 ZnO  2500 ZnO | *Blautia producta*  *Eubacterium plexicaudatum*  *unclassified Roseburia sp. 7*  *Terrisporobacter othiniensis*  *Holdemania filiformis*  *unclassified Eubacterium sp. 1*  *unclassified Oscillibacter sp. 1*  *Faecalitalea cylindroides*  *unclassified Clostridium sp. 14*  *unclassified Eubacterium sp. 2* |
| 110 ZnO  110 ZnLys | *Desulfovibrio piger*  *unclassified Ruminococcus sp. 6* |
| 2500 ZnO  110 ZnLys | *unclassified Prevotella sp. 18*  *Clostridium scindens*  *Bacteroides vulgatus* |
| 40 ZnO  110 ZnO  2500 ZnO | *Clostridium bornimense*  *Bacteroides coprocola*  *Bacteroides stercorirosoris*  *Prevotella bryantii*  *unclassified Prevotella sp. 8*  *Prevotella brevis*  *Cellulomonas carbonis*  *unclassified Eubacterium sp. 3*  *Sharpea azabuensis*  *Prevotella dentalis*  *unclassified Prevotella sp. 12* |
| 40 ZnO  110 ZnO  110 ZnLys | *unclassified Clostridium sp. 33*  *Acidaminococcus intestini*  *Treponema succinifaciens*  *unclassified Bacillus sp.*  *unclassified Clostridium sp. 25*  *unclassified Clostridium sp. 36*  *Ruminococcus champanellensis*  *unclassified Clostridium sp. 47*  *Lactobacillus amylolyticus*  *unclassified Ruminococcus sp. 5*  *unclassified Clostridium sp. 19*  *unclassified Clostridium sp. 39*  *unclassified Eubacterium sp. 5*  *unclassified Mycoplasma sp. 1*  *Acidaminococcus fermentans*  *unclassified Clostridium sp. 16*  *Ruminococcus bicirculans*  *unclassified Clostridium sp. 46*  *unclassified Clostridium sp. 9*  *unclassified Mycoplasma sp. 2*  *unclassified Clostridium sp. 30*  *unclassified Prevotella sp. 19*  *unclassified Mycoplasma sp. 3*  *Lactobacillus vaginalis*  *unclassified Clostridium sp. 17*  *Ruminococcus callidus*  *unclassified Megasphaera sp.*  *Megasphaera elsdenii*  *unclassified Clostridium sp. 23*  *unclassified Clostridium sp. 8*  *unclassified Faecalibacterium sp. 1*  *Prevotella maculosa*  *unclassified Oscillibacter sp. 3*  *unclassified Clostridium sp. 6*  *unclassified Ruminococcus sp. 7*  *unclassified Dialister sp.*  *unclassified Clostridium sp. 11*  *unclassified Ruminococcus sp. 8*  *Streptococcus infantarius*  *unclassified Clostridium sp. 34*  *unclassified Clostridium sp. 22*  *unclassified Clostridium sp. 31*  *unclassified Clostridium sp. 38*  *unclassified Clostridium sp. 5*  *unclassified Clostridium sp. 35*  *unclassified Clostridium sp. 28* |
