# Supplementary material for: Concentration and chemical form of dietary zinc shape the porcine colon microbiome, its functional capacity and antibiotic resistance gene repertoire
Source: ISME J. 2020 Aug 3;14(11):2783–93. doi: 10.1038/s41396-020-0730-3 (PMC7784847; doi:10.1038/s41396-020-0730-3)
Supplement: Supplementary file 7 — Supplemental Table S7 [file 41396_2020_730_MOESM7_ESM.docx]

**Supplemental Table S7**. Relative abundance of identified bacterial species that contribute to the discrimination of piglets fed diets with added zinc oxide at 40 ppm (40 ZnO), 110 ppm (110 ZnO), 2500 ppm (2500ZnO), or 110 ppm Zn-Lysinate (110ZnLys) as determined by Partial Least Squares-Discriminant Analysis (PLS-DA) and Variable Importance in Projection (VIP) scoring (n= 6/group).

|  |  | VIP score^1^ | 40 ZnO | 110 ZnO | 2500 ZnO | 110 ZnLys |
| --- | --- | --- | --- | --- | --- | --- |
| ***Genus*** | ***Species name*** |  | % of aligned sequences | | | |
| *Anaerotruncus* | *unclassified Anaerotruncus sp.* |  | 0.008 ± 0.004 | 0.043 ± 0.020 | 0.007 ± 0.003 | 0.011 ± 0.008 |
| *Bacteroides* | *Bacteroides barnesiae* | 1.104 | n.d. | n.d. | 0.013 ± 0.003 | n.d. |
|  | *Bacteroides faecichinchillae* | 1.042 | n.d. | 0.003 ± 0.003 | 0.088 ± 0.023 | 0.003 ± 0.003 |
|  | *Bacteroides faecis* | 1.200 | 0.023 ± 0.012 | 0.008 ± 0.005 | 0.005 ± 0.005 | 0.002 ± 0.002 |
|  | *Bacteroides gallinarum* | 1.020 | n.d. | n.d. | 0.057 ± 0.016 | n.d. |
|  | *Bacteroides thetaiotaomicron* | 1.075 | 0.004 ± 0.004 | n.d. | 0.043 ± 0.009 | n.d. |
| *Bifidobacterium* | *Bifidobacterium pseudocatenulatum* | 1.177 | n.d. | 0.018 ± 0.009 | 0.009 ± 0.003 | 0.004 ± 0.003 |
|  | *Bifidobacterium thermacidophilum* | 1.152 | 0.008 ± 0.006 | 0.045 ± 0.020 | 0.004 ± 0.004 | 0.005 ± 0.005 |
|  | *Bifidobacterium thermophilum* | 1.146 | 0.007 ± 0.005 | 0.050 ± 0.023 | 0.005 ± 0.005 | 0.009 ± 0.006 |
| *Blautia* | *Blautia schinkii* | 1.001 | 0.005 ± 0.003 | 0.012 ± 0.004 | 0.043 ± 0.009 | 0.003 ± 0.003 |
|  | *unclassified Blautia sp. 1* | 1.170 | n.d. | n.d. | 0.010 ± 0.003 | n.d. |
|  | *unclassified Blautia sp. 4* | 1.194 | n.d. | 0.006 ± 0.003 | 0.002 ± 0.002 | 0.007 ± 0.004 |
|  | *unclassified Blautia sp. 5* | 1.030 | 0.006 ± 0.006 | 0.013 ± 0.004 | 0.072 ± 0.015 | 0.004 ± 0.004 |
|  | *Blautia wexlerae* | 1.081 | 0.002 ± 0.002 | n.d. | 0.025 ± 0.005 | 0.004 ± 0.004 |
| *Cellulomonas* | *Cellulomonas carbonis* | 1.163 | 0.029 ± 0.009 | 0.005 ± 0.003 | 0.043 ± 0.027 | n.d. |
| *Chlamydia* | *Chlamydia suis* | 1.020 | 0.010 ± 0.008 | 0.146 ± 0.089 | 0.041 ± 0.037 | 0.003 ± 0.003 |
| *Clostridium* | *Clostridium clostridioforme* | 1.137 | n.d. | 0.003 ± 0.003 | 0.027 ± 0.008 | n.d. |
|  | *unclassified Clostridium sp. 10* | 1.185 | 0.048 ± 0.030 | 0.706 ± 0.411 | 0.517 ± 0.126 | 0.013 ± 0.006 |
|  | *unclassified Clostridium sp. 13* | 1.140 | 0.018 ± 0.007 | 0.013 ± 0.012 | n.d. | n.d. |
|  | *unclassified Clostridium sp. 14* | 1.078 | n.d. | 0.026 ± 0.016 | 0.020 ± 0.007 | n.d. |
|  | *unclassified Clostridium sp. 17* | 1.301 | 0.037 ± 0.028 | 0.329 ± 0.124 | n.d. | 0.073 ± 0.050 |
|  | *unclassified Clostridium sp. 18* | 1.337 | 0.008 ± 0.004 | 0.019 ± 0.006 | 0.011 ± 0.004 | 0.054 ± 0.024 |
|  | *unclassified Clostridium sp. 20* | 1.280 | 0.030 ± 0.015 | 0.137 ± 0.030 | 0.057 ± 0.028 | 0.068 ± 0.029 |
|  | *unclassified Clostridium sp. 22* | 1.280 | 0.006 ± 0.004 | 0.027 ± 0.010 | n.d. | 0.004 ± 0.003 |
|  | *unclassified Clostridium sp. 40* | 1.137 | 0.028 ± 0.010 | 0.093 ± 0.025 | 0.081 ± 0.011 | 0.042 ± 0.018 |
|  | *unclassified Clostridium sp. 42* | 1.345 | 0.002 ± 0.002 | 0.026 ± 0.009 | 0.005 ± 0.003 | 0.007 ± 0.004 |
|  | *unclassified Clostridium sp. 47* | 1.139 | 0.002 ± 0.002 | 0.017 ± 0.009 | n.d. | 0.003 ± 0.003 |
|  | *unclassified Clostridium sp. 48* | 1.083 | n.d. | n.d. | 0.020 ± 0.005 | n.d. |
|  | *unclassified Clostridium sp. 49* | 1.104 | 0.007 ± 0.003 | 0.022 ± 0.008 | 0.020 ± 0.004 | 0.007 ± 0.005 |
| *Desulfovibrio* | *Desulfovibrio piger* | 1.597 | n.d. | 0.009 ± 0.004 | n.d. | 0.040 ± 0.019 |
| *Dialister* | *Dialister succinatiphilus* | 1.788 | 0.546 ± 0.377 | 1.211 ± 1.057 | 0.036 ± 0.031 | 5.537 ± 1.454 |
| *Eggerthella* | *unclassified Eggerthella sp.* | 1.108 | 0.017 ± 0.005 | 0.041 ± 0.011 | 0.037 ± 0.010 | 0.013 ± 0.009 |
| *Eubacterium* | *Eubacterium desmolans* | 1.465 | n.d. | 0.009 ± 0.003 | 0.007 ± 0.003 | 0.009 ± 0.003 |
|  | *Eubacterium eligens* | 1.134 | 0.019 ± 0.007 | 0.008 ± 0.006 | n.d. | 0.102 ± 0.062 |
|  | *Eubacterium ramulus* | 1.158 | 0.005 ± 0.003 | 0.018 ± 0.005 | 0.017 ± 0.006 | 0.002 ± 0.002 |
| *Faecalibacterium* | *unclassified Faecalibacterium sp. 2* | 1.106 | 0.004 ± 0.003 | 0.003 ± 0.003 | 0.026 ± 0.005 | 0.002 ± 0.002 |
| *Faecalitalea* | *Faecalitalea cylindroides* | 1.021 | n.d. | 0.005 ± 0.003 | 0.036 ± 0.009 | n.d. |
| *Lactobacillus* | *Lactobacillus amylolyticus* | 1.068 | 0.002 ± 0.002 | 0.009 ± 0.006 | n.d. | 0.012 ± 0.004 |
|  | *Lactobacillus kitasatonis* | 1.338 | 0.039 ± 0.013 | 0.075 ± 0.031 | 0.046 ± 0.025 | 0.114 ± 0.022 |
|  | *Lactobacillus ultunensis* | 1.330 | 0.011 ± 0.004 | 0.026 ± 0.011 | 0.006 ± 0.006 | 0.041 ± 0.011 |
|  | *Lactobacillus vaginalis* | 1.264 | 0.013 ± 0.007 | 0.002 ± 0.002 | n.d. | 0.003 ± 0.003 |
| *Marvinbryantia* | *Marvinbryantia formatexigens* | 1.286 | n.d. | n.d. | 0.023 ± 0.002 | n.d. |
| *Mycoplasma* | *unclassified Mycoplasma sp. 3* | 1.346 | 0.032 ± 0.032 | 0.330 ± 0.150 | n.d. | 0.004 ± 0.004 |
| *Oscillibacter* | *unclassified Oscillibacter sp. 2* | 1.051 | 0.004 ± 0.002 | 0.010 ± 0.003 | 0.040 ± 0.008 | 0.002 ± 0.002 |
| *Prevotella* | *Prevotella dentasini* | 1.101 | n.d. | n.d. | 0.029 ± 0.007 | n.d. |
|  | *Prevotella maculosa* | 1.165 | 0.037 ± 0.024 | 0.014 ± 0.006 | n.d. | 0.002 ± 0.002 |
|  | *unclassified Prevotella sp. 1* | 1.066 | n.d. | n.d. | 0.045 ± 0.012 | n.d. |
|  | *unclassified Prevotella sp. 4* | 1.109 | n.d. | n.d. | 0.011 ± 0.003 | n.d. |
|  | *unclassified Prevotella sp. 10* | 1.185 | 2.225 ± 1.091 | 0.547 ± 0.181 | 0.323 ± 0.114 | 0.636 ± 0.402 |
|  | *unclassified Prevotella sp. 11* | 1.326 | 0.795 ± 0.239 | 0.430 ± 0.117 | 0.158 ± 0.042 | 0.344 ± 0.093 |
|  | *unclassified Prevotella sp. 13* | 1.485 | 2.798 ± 1.054 | 0.107 ± 0.025 | 1.223 ± 0.662 | 0.466 ± 0.201 |
|  | *unclassified Prevotella sp. 14* | 1.290 | 1.470 ± 0.960 | 0.005 ± 0.003 | 0.031 ± 0.008 | 0.003 ± 0.003 |
|  | *unclassified Prevotella sp. 18* | 1.027 | n.d. | n.d. | 0.064 ± 0.018 | 0.002 ± 0.002 |
|  | *unclassified Prevotella sp. 23* | 1.205 | 0.082 ± 0.020 | 0.044 ± 0.014 | 0.101 ± 0.016 | 0.035 ± 0.011 |
| *Ruminococcus* | *Ruminococcus gnavus* | 1.074 | n.d. | n.d. | 0.015 ± 0.004 | n.d. |
|  | *Ruminococcus bromii* | 1.233 | 0.007 ± 0.003 | 0.017 ± 0.011 | 0.008 ± 0.004 | 0.045 ± 0.026 |
|  | *Ruminococcus lactaris* | 1.128 | 0.010 ± 0.003 | 0.021 ± 0.007 | 0.046 ± 0.004 | 0.005 ± 0.004 |
|  | *unclassified Ruminococcus sp. 3* | 1.254 | 0.035 ± 0.005 | 0.090 ± 0.021 | 0.080 ± 0.011 | 0.052 ± 0.013 |
|  | *unclassified Ruminococcus sp. 5* | 1.385 | 0.720 ± 0.272 | 4.215 ± 1.525 | n.d. | 0.696 ± 0.326 |
| *Treponema* | *Treponema succinifaciens* | 1.361 | 0.278 ± 0.073 | 0.600 ± 0.165 | n.d. | 0.151 ± 0.083 |

n.d. - not determined

^1^VIP scores >1.0 are indicative of a major contribution to the discrimination of groups.
