# Supplementary material for: Concentration and chemical form of dietary zinc shape the porcine colon microbiome, its functional capacity and antibiotic resistance gene repertoire
Source: ISME J. 2020 Aug 3;14(11):2783–93. doi: 10.1038/s41396-020-0730-3 (PMC7784847; doi:10.1038/s41396-020-0730-3)
Supplement: Supplementary file 8 — Supplemental Table S8 [file 41396_2020_730_MOESM8_ESM.docx]

**Supplemental Table S8**. Relative abundance of GO slim terms related to biological process obtained from metagenomic sequences of microbial communities in colon digesta of piglets fed diets with added zinc oxide at 40 ppm (40 ZnO), 110 ppm (110 ZnO), 2500 ppm (2500ZnO), or 110 ppm Zn-Lysinate (110ZnLys) over a period of three weeks. Different superscripts indicate significant (P<0.05) differences (n= 6/group).

|  |  | 40 ZnO | 110 ZnO | 2500 ZnO | 110 ZnLys | SE | P-Value |
| --- | --- | --- | --- | --- | --- | --- | --- |
| **GO term** | **Description** | % of aligned sequences | | | |  |  |
| GO:0008152 | metabolic process | 12.82 | 12.63 | 13.14 | 13.00 | 0.221 | 0.116 |
| GO:0044281 | small molecule metabolic process | 12.02 | 11.86 | 11.86 | 12.04 | 0.157 | 0.486 |
| GO:0009058 | biosynthetic process | 11.02 | 10.43 | 10.81 | 10.61 | 0.265 | 0.165 |
| GO:0006810 | transport | 8.69^ab^ | 8.29^a^ | 9.28^b^ | 8.79^ab^ | 0.370 | 0.045 |
| GO:0006807 | nitrogen compound metabolic process | 8.99 | 8.46 | 8.84 | 8.91 | 0.262 | 0.203 |
| GO:0005975 | carbohydrate metabolic process | 8.34^b^ | 7.54^a^ | 7.45^a^ | 9.80^c^ | 0.371 | <0.001 |
| GO:0006259 | DNA metabolic process | 6.94^ab^ | 7.50^b^ | 7.26^ab^ | 6.58^a^ | 0.354 | 0.050 |
| GO:0006412 | translation | 5.78^ab^ | 6.41^b^ | 5.76^a^ | 5.39^a^ | 0.238 | 0.002 |
| GO:0016070 | RNA metabolic process | 5.37^a^ | 6.10^b^ | 5.48^a^ | 4.99^a^ | 0.232 | 0.001 |
| GO:0019222 | regulation of metabolic process | 2.79 | 2.89 | 2.90 | 2.86 | 0.107 | 0.709 |
| GO:0006508 | proteolysis | 2.66^ab^ | 2.59^ab^ | 2.52^a^ | 2.74^b^ | 0.088 | 0.042 |
| GO:0006950 | response to stress | 1.94^b^ | 2.02^b^ | 1.98^b^ | 1.68^a^ | 0.065 | <0.001 |
| GO:0016310 | phosphorylation | 1.67 | 1.73 | 1.67 | 1.73 | 0.039 | 0.117 |
| GO:0006351 | transcription, DNA-templated | 1.66^ab^ | 1.78^b^ | 1.58^a^ | 1.58^a^ | 0.062 | 0.008 |
| GO:0071840 | cellular component organization or biogenesis | 1.56^ab^ | 1.72^b^ | 1.62^ab^ | 1.45^a^ | 0.060 | 0.001 |
| GO:0006629 | lipid metabolic process | 1.54^ab^ | 1.47^a^ | 1.50^ab^ | 1.57^b^ | 0.020 | <0.001 |
| GO:0071103 | DNA conformation change | 1.32 | 1.49 | 1.36 | 1.31 | 0.083 | 0.120 |
| GO:0000160 | phosphorelay signal transduction system | 0.96^a^ | 0.99^a^ | 0.92^a^ | 1.11^b^ | 0.009 | 0.002 |
| GO:0006091 | generation of precursor metabolites and energy | 0.77 | 0.79 | 0.80 | 0.75 | 0.026 | 0.355 |
| GO:0008150 | biological process | 0.65^ab^ | 0.83^b^ | 0.75^ab^ | 0.63^a^ | 0.070 | 0.034 |
| GO:0006457 | protein folding | 0.67 | 0.69 | 0.65 | 0.67 | 0.023 | 0.276 |
| GO:0007165 | signal transduction | 0.43^ab^ | 0.39^ab^ | 0.38^a^ | 0.51^b^ | 0.053 | 0.009 |
| GO:0042221 | response to chemical | 0.33 | 0.31 | 0.32 | 0.32 | 0.026 | 0.915 |
| GO:0045454 | cell redox homeostasis | 0.18 | 0.19 | 0.19 | 0.20 | 0.013 | 0.412 |
| GO:0016226 | iron-sulfur cluster assembly | 0.17^b^ | 0.18^b^ | 0.17^b^ | 0.14^a^ | 0.032 | 0.002 |
| GO:0043934 | sporulation | 0.09^a^ | 0.20^b^ | 0.11^ab^ | 0.12^ab^ | 0.034 | 0.028 |
| GO:0071973 | bacterial-type flagellar cell motility | 0.17 | 0.10 | 0.13 | 0.11 | 0.032 | 0.134 |
| GO:0016032 | viral process | 0.08 | 0.06 | 0.10 | 0.07 | 0.018 | 0.145 |
| GO:0009405 | pathogenesis | 0.07^ab^ | 0.05^ab^ | 0.10^b^ | 0.02^a^ | 0.021 | 0.006 |
| GO:0009607 | response to biotic stimulus | 0.07 | 0.06 | 0.07 | 0.06 | 0.007 | 0.166 |
| GO:0009372 | quorum sensing | 0.06 | 0.05 | 0.06 | 0.04 | 0.004 | 0.075 |
| GO:0065003 | macromolecular complex assembly | 0.05 | 0.06 | 0.06 | 0.04 | 0.008 | 0.051 |
| GO:0006808 | regulation of nitrogen utilization | 0.06 | 0.04 | 0.06 | 0.05 | 0.009 | 0.158 |
| GO:0006282 | regulation of DNA repair | 0.03 | 0.03 | 0.03 | 0.03 | 0.002 | 0.120 |
| GO:0017004 | cytochrome complex assembly | 0.03 | 0.02 | 0.03 | 0.03 | 0.006 | 0.355 |
| GO:0007155 | cell adhesion | 0.01 | 0.01 | 0.04 | 0.02 | 0.023 | 0.390 |
| GO:0015948 | methanogenesis | 0.01 | 0.01 | 0.02 | 0.00 | 0.005 | 0.095 |
