# Supplementary material for: Concentration and chemical form of dietary zinc shape the porcine colon microbiome, its functional capacity and antibiotic resistance gene repertoire
Source: ISME J. 2020 Aug 3;14(11):2783–93. doi: 10.1038/s41396-020-0730-3 (PMC7784847; doi:10.1038/s41396-020-0730-3)
Supplement: Supplementary file 9 — Supplemental Table S9 [file 41396_2020_730_MOESM9_ESM.docx]

**Supplemental Table S9**. Relative abundance of GO slim terms related to molecular function obtained from metagenomic sequences of microbial communities in colon digesta of piglets fed diets with added zinc oxide at 40 ppm (40 ZnO), 110 ppm (110 ZnO), 2500 ppm (2500ZnO), or 110 ppm Zn-Lysinate (110ZnLys) over a period of three weeks. Different superscripts indicate significant (P<0.05) differences (n= 6/group).

|  |  | 40 ZnO | 110 ZnO | 2500 ZnO | 110 ZnLys | SE | P-Value |
| --- | --- | --- | --- | --- | --- | --- | --- |
| **GO term** | **Description** | % of aligned sequences | | | |  |  |
| GO:0000166 | nucleotide binding | 14.56^ab^ | 15.68^b^ | 14.86^ab^ | 14.00^a^ | 0.257 | 0.003 |
| GO:0003676 | nucleic acid binding | 12.23 | 12.91 | 12.37 | 12.02 | 0.204 | 0.155 |
| GO:0003824 | catalytic activity | 11.70^ab^ | 10.91^a^ | 11.42^ab^ | 11.94^b^ | 0.198 | 0.046 |
| GO:0016740 | transferase activity | 7.26^ab^ | 7.29^b^ | 7.49^b^ | 7.02^a^ | 0.065 | 0.001 |
| GO:0016787 | hydrolase activity | 6.75^a^ | 6.37^a^ | 6.38^a^ | 7.40^b^ | 0.148 | <0.001 |
| GO:0016491 | oxidoreductase activity | 6.35^ab^ | 6.30^ab^ | 6.69^b^ | 6.27^a^ | 0.084 | 0.033 |
| GO:0016874 | ligase activity | 4.57^a^ | 5.03^b^ | 4.64^a^ | 4.39^a^ | 0.094 | 0.001 |
| GO:0005215 | transporter activity | 4.00 | 3.68 | 4.37 | 3.89 | 0.136 | 0.062 |
| GO:0016853 | isomerase activity | 3.46^ab^ | 3.53^ab^ | 3.36^a^ | 3.65^b^ | 0.043 | 0.001 |
| GO:0017111 | nucleoside-triphosphatase activity | 3.26^a^ | 3.65^b^ | 3.42^ab^ | 3.17^a^ | 0.078 | 0.006 |
| GO:0008233 | peptidase activity | 2.45 | 2.40 | 2.34 | 2.50 | 0.034 | 0.144 |
| GO:0003735 | structural constituent of ribosome | 2.15^ab^ | 2.26^b^ | 2.08^ab^ | 1.97^a^ | 0.045 | 0.004 |
| GO:0016829 | lyase activity | 2.08^ab^ | 1.90^a^ | 2.07^ab^ | 2.15^b^ | 0.046 | 0.035 |
| GO:0050662 | coenzyme binding | 2.09^ab^ | 1.95^a^ | 2.12^b^ | 2.01^ab^ | 0.031 | 0.025 |
| GO:0016301 | kinase activity | 1.77 | 1.82 | 1.76 | 1.82 | 0.015 | 0.070 |
| GO:0005515 | protein binding | 1.91^ab^ | 1.60^a^ | 1.57^ab^ | 2.02^b^ | 0.085 | 0.007 |
| GO:0016779 | nucleotidyltransferase activity | 1.73^ab^ | 1.81^b^ | 1.72^ab^ | 1.66^a^ | 0.027 | 0.042 |
| GO:0003700 | transcription factor activity, sequence-specific DNA binding | 1.21 | 1.22 | 1.22 | 1.25 | 0.012 | 0.602 |
| GO:0004872 | receptor activity | 1.19^ab^ | 0.82^a^ | 0.87^a^ | 1.57^b^ | 0.122 | 0.002 |
| GO:0003674 | molecular function | 0.83 | 0.73 | 0.75 | 0.77 | 0.020 | 0.090 |
| GO:0004803 | transposase activiy | 0.80^b^ | 0.70^ab^ | 0.85^b^ | 0.57^a^ | 0.040 | <0.001 |
| GO:0030170 | pyridoxal phosphate binding | 0.72 | 0.61 | 0.70 | 0.71 | 0.028 | 0.170 |
| GO:0030246 | carbohydrate binding | 0.60^bc^ | 0.44^a^ | 0.48^ab^ | 0.64^c^ | 0.031 | <0.001 |
| GO:0051536 | iron-sulfur cluster binding | 0.50 | 0.50 | 0.50 | 0.49 | 0.010 | 0.938 |
| GO:0004871 | signal transducer activity | 0.49^ab^ | 0.46^a^ | 0.45^a^ | 0.58^b^ | 0.024 | 0.012 |
| GO:0019842 | vitamin binding | 0.47^ab^ | 0.41^a^ | 0.46^ab^ | 0.55^b^ | 0.022 | 0.006 |
| GO:0000150 | recombinase activity | 0.33 | 0.47 | 0.40 | 0.35 | 0.034 | 0.160 |
| GO:0016597 | amino acid binding | 0.32 | 0.31 | 0.32 | 0.32 | 0.006 | 0.714 |
| GO:0046906 | tetrapyrrole binding | 0.24^ab^ | 0.21^a^ | 0.24^ab^ | 0.32^b^ | 0.021 | 0.023 |
| GO:0008658 | penicillin binding | 0.24^a^ | 0.30^ab^ | 0.51^b^ | 0.23^a^ | 0.048 | 0.006 |
| GO:0016791 | phosphatase activity | 0.24 | 0.19 | 0.23 | 0.26 | 0.014 | 0.074 |
| GO:0090484 | drug transporter activity | 0.20 | 0.22 | 0.20 | 0.22 | 0.008 | 0.306 |
| GO:0008134 | transcription factor binding | 0.11^ab^ | 0.08^a^ | 0.11^ab^ | 0.13^b^ | 0.009 | 0.020 |
| GO:0016209 | antioxidant activity | 0.08 | 0.07 | 0.11 | 0.10 | 0.012 | 0.711 |
| GO:0009055 | electron carrier activity | 0.07 | 0.07 | 0.08 | 0.07 | 0.006 | 0.208 |
| GO:0043167 | ion binding | 0.03 | 0.02 | 0.02 | 0.02 | 0.001 | 0.084 |
