# Supplementary material for: Concentration and chemical form of dietary zinc shape the porcine colon microbiome, its functional capacity and antibiotic resistance gene repertoire
Source: ISME J. 2020 Aug 3;14(11):2783–93. doi: 10.1038/s41396-020-0730-3 (PMC7784847; doi:10.1038/s41396-020-0730-3)
Supplement: Supplementary file 10 — Supplemental Table S10 [file 41396_2020_730_MOESM10_ESM.docx]

**Supplemental Table S10**. Relative abundance of antibiotic resistance genes (given as number of aligned reads per 1000bp of reference gene) in colon digesta of piglets fed diets with added zinc oxide at 40 ppm (40 ZnO), 110 ppm (110 ZnO), 2500 ppm (2500ZnO), or 110 ppm Zn-Lysinate (110ZnLys) over a period of three weeks. Different superscripts indicate significant (P<0.05) differences (n= 6/group).

|  |  | 40 ZnO | 110 ZnO | 2500 ZnO | 110 ZnLys | SE | P-Value |
| --- | --- | --- | --- | --- | --- | --- | --- |
| **Antibiotic class** | **Gene symbol** | reads per 1000bp of reference gene | | | |  |  |
| Aminoglycosides | *aac(6')-Ip* | 2 | 7 | 3 | 9 | 4.1 | 0.281 |
|  | *ant(6)-Ia* | 27 | 57 | 14 | 35 | 22.3 | 0.307 |
|  | *ant(6)-Ib* | 535 | 832 | 761 | 677 | 189.9 | 0.454 |
|  | *ant(9)-Ia* | 8 | 32 | 0 | 17 | 16.6 | 0.292 |
|  | *aph(2'')-IIa* | 2 | 10 | 7 | 8 | 4.6 | 0.399 |
|  | *aph(3'')-Ib* | 13^ab^ | 1^a^ | 35^b^ | 5^a^ | 9.3 | 0.007 |
|  | *aph(3')-IIIa* | 117 | 179 | 151 | 273 | 57.1 | 0.069 |
| β-Lactams | *blaROB* | 10^a^ | 1^a^ | 72^b^ | 2^a^ | 18.8 | 0.003 |
|  | *cfxA2* | 233^b^ | 82^a^ | 140^ab^ | 139^ab^ | 55.9 | 0.092 |
|  | *cfxA5* | 20 | 6 | 12 | 9 | 4.9 | 0.042 |
|  | *cfxA6* | 550 | 243 | 391 | 136 | 220.9 | 0.292 |
| Fluoroquinolones | *pat(A)* | 11^a^ | 0^a^ | 117^b^ | 24^a^ | 37.1 | 0.049 |
| Glycopeptides | *vanB gene cluster* | 9 | 37 | 14 | 13 | 20.6 | 0.531 |
| Lincosamides | *lnu(C)* | 745^ab^ | 495^ab^ | 1037^b^ | 157^a^ | 221.9 | 0.043 |
| Macrolides | *erm(B)* | 15 | 5 | 103 | 32 | 63.1 | 0.422 |
|  | *erm(F)* | 56 | 17 | 15 | 37 | 17.9 | 0.114 |
|  | *erm(G)* | 24^a^ | 24^a^ | 23^a^ | 78^b^ | 19.0 | 0.019 |
|  | *erm(T)* | 38 | 53 | 25 | 52 | 48.2 | 0.161 |
|  | *mef(A)* | 137 | 76 | 93 | 167 | 38.4 | 0.108 |
| Polypeptides | *arnA* | 7^a^ | 0^a^ | 126^b^ | 21^a^ | 21.3 | 0.041 |
|  | *bac(A)* | 12 | 1 | 53 | 16 | 37.1 | 0.543 |
|  | *pmrB* | 10 | 0 | 86 | 25 | 55.6 | 0.429 |
|  | *pmrC* | 12 | 0 | 143 | 25 | 92.1 | 0.398 |
|  | *pmrE* | 4 | 0 | 29 | 23 | 23.5 | 0.565 |
|  | *pmrF* | 7 | n.d. | 47 | 16 | 32.2 | 0.488 |
| Streptothricin | *sat4* | 68 | 76 | 76 | 109 | 22.0 | 0.271 |
| Sulfonamides | *sul2* | 0 | n.d. | 1 | 0 | 0.4 | 0.051 |
| Tetracyclines | *tet(32)* | 163 | 165 | 119 | 104 | 52.1 | 0.550 |
|  | *tet(40)* | 315 | 392 | 333 | 455 | 78.6 | 0.300 |
|  | *tet(44)* | 212 | 686 | 503 | 188 | 219.0 | 0.098 |
|  | *tet(L)* | 114 | 129 | 26 | 1 | 77.7 | 0.292 |
|  | *tet(O)* | 275 | 262 | 276 | 260 | 61.4 | 0.990 |
|  | *tet(Q)* | 2129 | 998 | 1327 | 2220 | 482.1 | 0.052 |
|  | *tet(W)* | 1532 | 1995 | 2269 | 1124 | 466.1 | 0.076 |
