# Supplementary material for: Concentration and chemical form of dietary zinc shape the porcine colon microbiome, its functional capacity and antibiotic resistance gene repertoire
Source: ISME J. 2020 Aug 3;14(11):2783–93. doi: 10.1038/s41396-020-0730-3 (PMC7784847; doi:10.1038/s41396-020-0730-3)
Supplement: Supplementary file 11 — Supplemental Figure S1 [file 41396_2020_730_MOESM11_ESM.docx]

**Supplemental Figure S1**. Relative abundance of GO terms ‘metal ion binding’ (GO:0046872) (**A**), ‘metal ion transport’(GO:0030001) (**B**), penicillin binding’ (GO: 0008658) (**C**) and ‘β-lactam catabolism’ (GO:0030655) (**D**) obtained from metagenomic sequences of microbial communities in colon digesta of piglets fed diets with added zinc oxide at 40 ppm (40 ZnO), 110 ppm (110 ZnO), 2500 ppm (2500ZnO), or 110 ppm Zn-Lysinate (110ZnLys) over a period of three weeks. Different superscripts indicate significant (P<0.05) differences (n= 6/group).

*
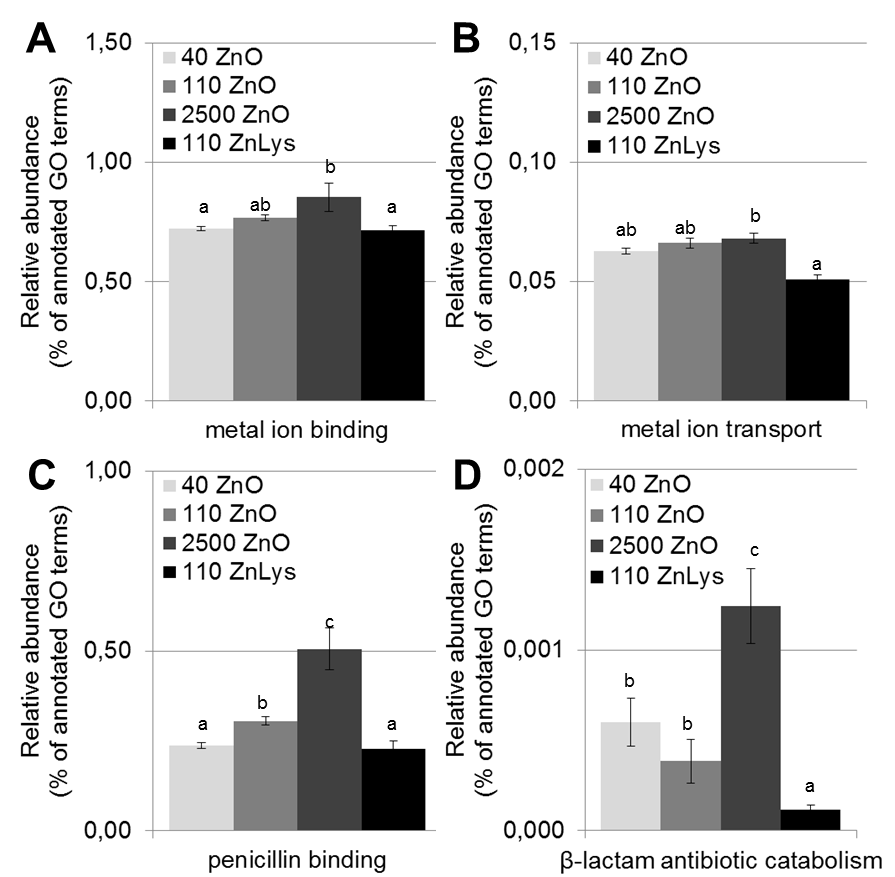
*
